# Pathogen stimulations and immune cells synergistically affect the gene expression profile characteristics of porcine peripheral blood mononuclear cells

**DOI:** 10.1186/s12864-024-10603-9

**Published:** 2024-07-25

**Authors:** Jinyan Yang, Siqian Chen, Fuping Ma, Ning Ding, Siyuan Mi, Qingyao Zhao, Yue Xing, Ting Yang, Kai Xing, Ying Yu, Chuduan Wang

**Affiliations:** 1 Key Laboratory of Animal Genetics, Breeding and Reproduction, Ministry of Agriculture & National Engineering Laboratory for Animal Breeding, College of Animal Science and Technologyn, China Agricultural University, Beijing, 100193 China; 2 Dabei-Nong Science and Technology Group Co., Ltd, Beijing, 100080 China

**Keywords:** Gene expression regulation, Pathogen stimulations, Peripheral blood mononuclear cells, Cell-specific expression, Context-dependent transcriptional response, Expression quantitative trait locus

## Abstract

**Background:**

Pigs serve as a crucial source of protein in the human diet and play a fundamental role in ensuring food security. However, infectious diseases caused by bacteria or viruses are a major threat to effective global pig farming, jeopardizing human health. Peripheral blood mononuclear cells (PBMCs) are a mixture of immune cells that play crucial roles in immunity and disease resistance in pigs. Previous studies on the gene expression regulation patterns of PBMCs have concentrated on a single immune stimulus or immune cell subpopulation, which has limited our comprehensive understanding of the mechanisms of the pig immune response.

**Results:**

Here, we integrated and re-analyzed RNA-seq data published online for porcine PBMC stimulated by lipopolysaccharide (LPS), polyinosinic acid (PolyI:C), and various unknown microorganisms (EM). The results revealed that gene expression and its functional characterization are highly specific to the pathogen, identifying 603, 254, and 882 pathogen-specific genes and 38 shared genes, respectively. Notably, LPS and PolyI:C stimulation directly triggered inflammatory and immune-response pathways, while exposure to mixed microbes (EM) enhanced metabolic processes. These pathogen-specific genes were enriched in immune trait-associated quantitative trait loci (QTL) and eGenes in porcine immune tissues and were implicated in specific cell types. Furthermore, we discussed the roles of eQTLs rs3473322705 and rs1109431654 in regulating pathogen- and cell-specific genes *CD300A* and *CD93,* using cellular experiments. Additionally, by integrating genome-wide association studies datasets from 33 complex traits and diseases in humans, we found that pathogen-specific genes were significantly enriched for immune traits and metabolic diseases.

**Conclusions:**

We systematically analyzed the gene expression profiles of the three stimulations and demonstrated pathogen-specific and cell-specific gene regulation across different stimulations in porcine PBMCs. These findings enhance our understanding of shared and distinct regulatory mechanisms of genetic variants in pig immune traits.

**Supplementary Information:**

The online version contains supplementary material available at 10.1186/s12864-024-10603-9.

## Introduction

Pigs play a crucial role in ensuring global food security as they are the main source of protein in the human diet. According to the National Bureau of Statistics of the People's Republic of China in 2022, pork production accounted for 59.4% of all meat production, and pig farming occupies a pivotal position in the country's agricultural sector. However, in recent years, infections caused by viruses (e.g., African swine fever virus) and bacteria (e.g., *Streptococcus suis*) have caused significant economic losses and animal health problems, greatly limiting the sustainable and healthy development of the global pig industry [1, 2]. Enhancing disease resistance is a potential key factor in preventing performance degradation due to health impairments. In general, disease resistance in pigs encompasses both general and specific resistance, both of which are equally crucial in the selection process. Specific resistance pertains to the body's targeted defense against specific bacterial or viral diseases, while general resistance is not confined to any particular pathogen and represents the overall robustness of the pig's defenses against disease [3, 4]. The key step in improving disease resistance is to dissect the host–pathogen interactions and host immune response pathways following infection.

Peripheral blood mononuclear cells (PBMCs) are one of the most important materials for functional immunological studies [5], which are composed of different combinations of immune cells, and serve as an effective in vitro model for the study of immunity and disease resistance traits in pigs [6]. Host immune response mechanisms vary according to pathogen and genetics. For example, our previous studies show that viral infection induces type I interferon signaling, which significantly upregulates the expression levels of cytokines such as *IL-10* and induces persistent host infection [7, 8]. Bacterial invasion immediately activates signaling pathways associated with inflammation to regulate the host immune response, while *IL10* expression is significantly down-regulated in the early stages of infection [9, 10]. Previous studies have used lipopolysaccharide (LPS) or polyinosinic acid (PolyI:C) to stimulate PBMCs in order to simulate the inflammatory response induced by bacteria or viruses, respectively, and found important genes, networks and signaling pathways related to immunity and disease resistance in pigs [11–14]. For example, Li et al. found that LPS stimulation induced a pronounced inflammatory response as evidenced by the upregulation of pro-inflammatory cytokines, chemokines, and related signaling pathways (e.g., NF-κB) [14]. However, most studies have only focused on the exploration of transcriptional mechanisms in pig PBMCs in response to a single stimulus, and there is still a lack of research on the similarities and differences between LPS and PolyI:C, as well as the transcriptional characterization of porcine-specific immune stimulation. Furthermore, the heterogeneity of different cell types in porcine PBMCs leads to different responses to different pathogens. Thus, under the premise of defining cell types using single-cell RNA sequencing (scRNA-seq), systematic analyses of the responses of different cells will significantly improve our understanding of the pathogenicity of various pathogens [6, 15–17]. However, the transcriptional response of different types of PBMCs in pigs infected with different pathogens remains incomplete, and the shared and differential immune cell effects of PBMCs in pigs have not been fully explored.

In this study, we uniformly integrated and re-analyzed the RNA-seq data of PBMCs after stimulation with three types of pathogen stimulations (LPS, PolyI:C, and multiple unknown pathogenic microorganisms (EM)) publicly available online, to characterize the gene regulation of immune responses to bacterial, viral, and various unknown pathogen stimuli, respectively. Our aim was to provide data to support the enhancement of pig-specific and general resistance to disease. Our comprehensive analysis revealed that gene expression and function exhibited profound specificity to the pathogen, with distinct biological processes being enriched across the three stimuli. Furthermore, we investigated the dynamic response of immune cell subpopulations in PBMCs to three different stimuli using strategies similar to single-cell analysis for the first time to quantify the proportions of the five major immune cells in PBMCs under different stimulations, reveals the critical role of monocytes in the immune response. Utilizing publicly accessible Pig quantitative trait locus (QTL) and PigGTEX datasets, we discovered that stimulus-specific genes exhibit a significant enrichment in QTL regions that are associated with immune traits, including the IFN-γ QTL and the monocyte count QTL. Meanwhile, we utilized a dataset of PRRSV-stimulated PBMCs to further validate that the PolyI:C stimulus-specific genes expression regulatory features of *CD300A* and *CD93*. Notably, the transcriptional levels of these genes are modulated by genetic variation. Also, our analysis revealed that bacterial and viral stimuli triggered transcriptional alterations in porcine immune cell subpopulations, with a remarkable enrichment of GWAS signals pertaining to human inflammatory and metabolic diseases. Specifically, genes specific to LPS and PolyI:C stimulation displayed a substantial enrichment of inflammation-related GWAS signals. Collectively, these findings underscore the analogous roles played by stimulus-specific genes in immune and metabolic diseases across pigs and humans, further reinforcing the potential of pigs as biological models for studying human diseases.

## Results

### PBMCs transcriptomes revealed pathogen-specific characteristics across three immune stimulus conditions

**Fig. 1:**
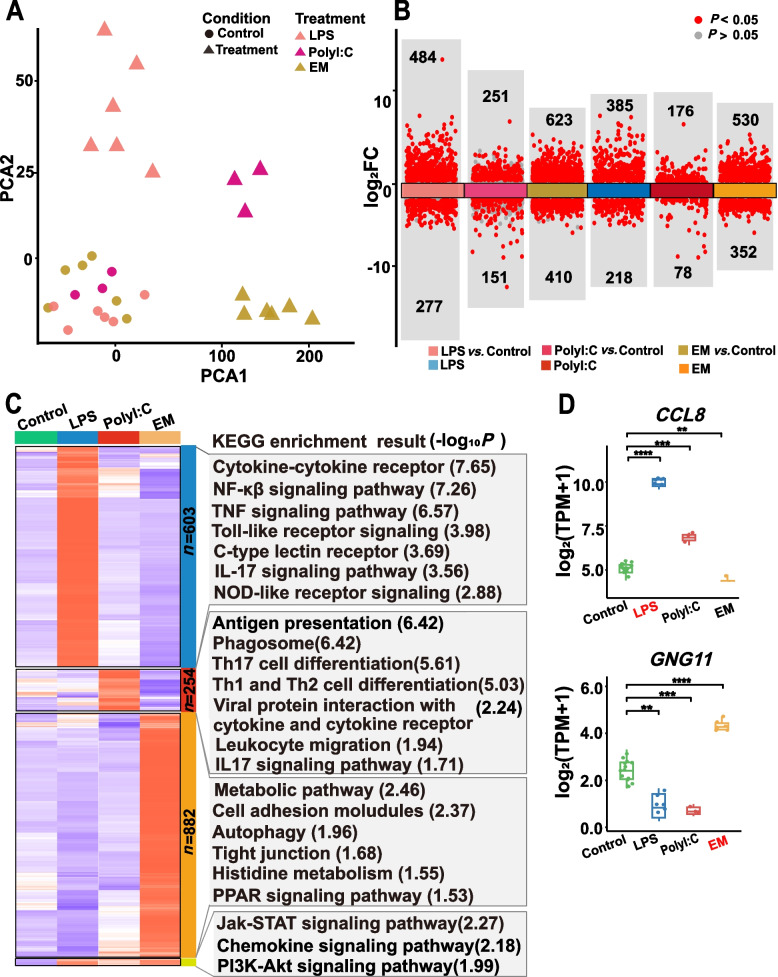
Gene expression profiles of pig peripheral blood mononuclear cells (PBMCs) with three stimulations (*n* = 30). **A** RNA-seq data for LPS-stimulated, PolyI:C-stimulated, EM-stimulated PBMCs and their controls, with different colors representing different types of stimulation and shapes indicating whether or not stimulation occurred. **B** The number of differentially expressed genes (DEGs) (*P* < 0.05, log_2_ (fold change) > 1.5) and specific genes with different stimulation. **C** Functional enrichment analysis was conducted on stimulus-specific DEGs and shared genes.** D** Examples of genes shared with different stimulations. Both *CCL8* and *GNG11* were shared between different stimuli, and their expression levels increased significantly after LPS stimulation and PolyI:C stimulation, respectively, compared to the other two stimulated groups

To characterize the gene expression patterns of PBMCs under bacterial, viral, and various unknown pathogen stimuli, we analyzed unstimulated and stimulated RNA-seq data using LPS [14], PolyI:C [18], and exposure to multiple pathogens and microbial environments (EM) (*n* = 30) [19]. PCA analyses of gene expression profiles revealed a strong difference in the stimulation effects (Figs. 1A, S3A). Compared to the untreated PBMC group, we identified 761, 1,033, and 402 differentially expressed genes (DEGs) after LPS, EM, and PolyI:C stimulation, respectively. We also found that the expression of more than half of these genes increased considerably (Fig. 1B). It is worth noting that only 0.9% of DGEs share in the three stimuli, and most of the genes are stimulus-specific genes, demonstrating the considerable context-dependence of gene expression. KEGG results indicated that the shared genes were mainly involved in the Jak-STAT, chemokine, and PI3K-Akt signaling pathways (Fig. 1C). In addition, approximately 106 DEGs were shared between the EM- and LPS-stimulated groups (Fig. S3C), and 71 DEGs were shared between the EM-and PolyI:C-stimulated groups, most likely because LPS is a crucial component of the outer membrane of gram-negative bacteria [20], making it a perfect chemical for the immune system to recognize bacteria. Notably, LPS and PolyI:C stimulation shared 95 DEGs, but more than 76.8% *(n* = 73) of these DEGs showed higher fold enrichment after LPS stimulation. These findings imply that hosts may respond to bacterial and viral infections using different immune response strategies and that during the initial phases of an infection, the bacteria-induced immune response is more potent than the viral-induced response. Finally, 603, 882, and 254 LPS-, EM-, and PolyI:C-specific genes, respectively, were identified, along with 18 shared genes.

To explore the function of specific genes in response to different stimuli, a functional enrichment analysis was performed using KEGG. Similar to the gene expression characteristics, stimulation-specific genes tended to be enriched in specific biological pathways (Fig. 1C, Supplemental Table S2). The results showed that the stimulation-specific genes of LPS and PolyI:C stimulations induced different specific immune responses compared to the EM-stimulation, which led to the over-representation of genes associated with immune receptor pathways, including cytokine-cytokine receptor interaction and NOD-like receptor signaling pathways, together with signal transduction pathways, including TNF, NF-κβ, and Toll-like signaling pathways (Fig. 1C, Supplemental Table S2). The specific adaptation of PBMCs to EM-stimulation was characterized by the dysregulation of tight intercellular junctions and increased metabolic activity (Fig. 1C, Supplemental Table S2). These results indicate the direct activation of pathways associated with immune activity following LPS and PolyI:C stimulation, whereas EM-stimulation may enhance metabolic pathways necessary for cellular processes. For example, the chemokine CCL8, a member of the CC chemokine family, is considered an important pro-inflammatory cytokine that alters the state of the body's immune system through the activation of various immune cells and is considered a key factor in maintaining the body's self-regulation [21]. The expression levels of *CCL8* significantly increased after LPS- and PolyI:C- stimulation (Fig. 1D). Another example is *GNG11*, whose expression was significantly upregulated after EM-stimulation and was enriched in amino acid metabolism and cell adhesion-related pathways (Fig. 1D). Collectively, these observations reveal the gene regulatory network of PBMCs under three stimulus conditions, suggesting that the host may adopt different immune response strategies in response to different stimuli.

### Identification of key modules and hub genes of immune activation and metabolic processes

**Fig. 2:**
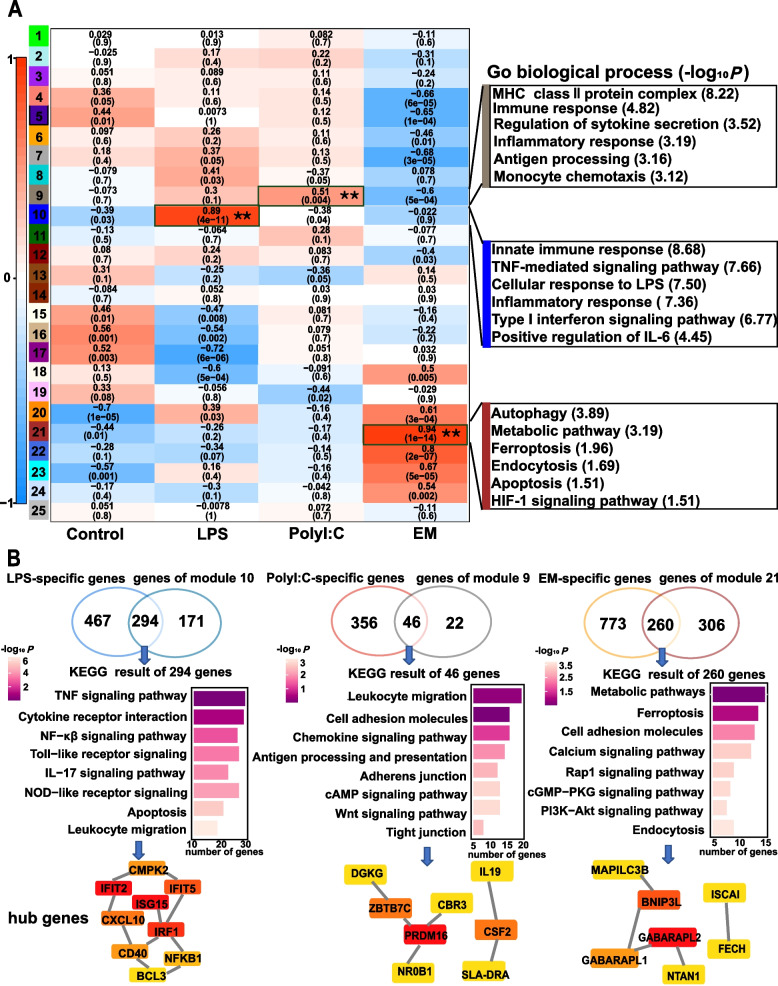
Weighted correlation network analysis (WGCNA) and differential expression integration analysis to screen candidate hub genes for different stimulations. **A** Heatmap showing the relationship between modules and samples (left panel), The x-axis represents the sample information, the y-axis represents each gene module, red indicates positive correlation, and blue indicates negative correlation. The right panel indicates the GO analysis results for the darkgrey, blue, and brown modules. “**” indicates modules significantly correlated with stimulus modality (FDR < 0.01). **B** Functional enrichment analysis of key genes and candidate hub genes with different stimulation

To identify key modules and hub genes associated with different stimuli, 30 RNA-seq datasets were analyzed using WGCNA (Fig. S4A). A total of 25 modules were derived from the WGCNA analysis, with the number of genes in each module ranging from 33 to 1,859. Next, modules 10, 9, and 21 were significantly associated (FDR < 0.01) with the LPS-, Poly I:C-, and EM-stimulated groups, respectively (FDR < 0.01) (Figs. 2A, S4B). For instance, the hub genes of module 10 were significantly involved in the innate immune and inflammatory responses. The hub genes of module 9 were primarily linked to immune response, monocyte phagocytosis, and the MHC II protein complex. The hub genes of module 21 were significantly associated with the regulation of cellular autophagy, ferroptosis, and metabolic processes (Fig. 2A).

A Venn plot was generated to search for candidate genes among the DEGs and hub genes (Fig. 2B, upper panel), and 294, 46, and 260 candidate genes in the LPS-, PolyI:C-, and EM-stimulated groups, respectively (Fig. 2B). As expected, the results showed that candidate genes of the LPS and PolyI:C stimulations were associated with immune activation signals, such as TNF, NF-κβ, leukocyte migration, and antigen presentation signaling pathways, while candidate genes of the EM stimulation were associated with substance metabolism as well as cell adhesion molecules (Fig. 2B, middle panel). Next, we identified the candidate genes in each module (Fig. 2B, lower panel), most of which were involved in the activation of immune responses and host metabolic processes. For example, *SLA-DRA*, located on the surface of antigen-presenting cells, was identified as a candidate gene of the PolyI:C-stimulated group (Fig. 2B). It plays a critical role in regulating and maintaining overall adaptive immune resistance to pathogens and has emerged as a target gene affecting resistance or susceptibility to disease in pigs [22, 23]. Furthermore, *GABARAPL1* and *GABARAPL2*, identified as candidate genes of the EM-stimulated group (Fig. 2B), were considered markers of the cellular autophagic process and were involved not only in protein or vesicle transport but also in autophagy, cell death, and cell proliferation [24].

### Stimulus-specific gene expression revealed immune cell-type specificity in PBMCs

Previous studies have reported that gene expression shows pathogen-specific and cell type-specific characteristics in humans and pigs, such as T cells, B cells, and monocytes (mono), in response to viral or bacterial stimulation of PBMCs [25]. To identify specific genes expressed in different immune cell types, we performed an integrative analysis using RNA-seq data from immune cells publicly available online [26–29]. The results of PCA showed that all immune cells were classified into T-cells, B-cells, cDCs, pDCs and monocytes based on gene expression differences (Fig. S3B). Cell specificity of gene expression was quantified using the tissue specificity index (TAU) (Fig. S5A, S5B). GO analysis revealed that many genes with specific functions were preferentially expressed in specific cell types (Fig. S6A, Supplemental Table S3). For example, pDC-specific genes were mainly involved in the MAPK pathway and cell adhesion processes, while monocyte-specific genes were mainly involved in immunity, inflammation, and phagocytosis of pathogens (Fig. S6A).

**Fig. 3:**
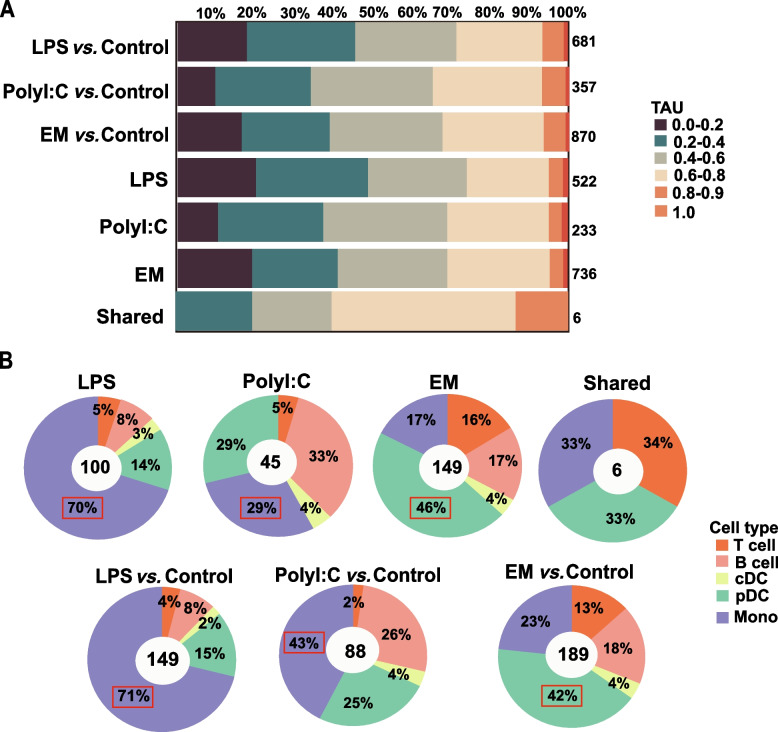
Proportion of immune cell types in PBMC with different stimuli. DEGs of PBMC with LPS stimulation (LPS *vs.* control), PolyI:C stimulation (PolyI:C *vs.* control) and EM stimulation (EM *vs.* control) compared to control, as well as the proportion of stimulus-specific DEGs (LPS, PolyI:C, and EM) and inter-stimulus shared DEGs (Shared) in the proportion of TAU values (**A**) and the proportion of each immune cell-specific genes (**B**)

Understanding the changes in immune cell subsets and states in pig PBMCs under different stimulations is essential for improving pig disease resistance. Thus, we assessed immune changes in pig PBMCs following LPS, PolyI:C, and EM stimulation, which induced a wide array of differential expression of cell-specific genes (Fig. 3A). Notably, LPS and PolyI:C induced similar immune response in porcine PBMCs, as shown by significant changes in the expression levels of most monocyte specific genes (29.0–70.0%) and comparatively minor changes in T-cell-specific genes expression (5.0%). This may be related to the activation of the host's innate immune response at the initial stage of bacterial and viral infections, further underscoring the key role of monocytes as antigen-presenting cells in the host's immune response [30]. Remarkably, EM-stimulation yielded a unique expression profile, characterized predominantly by a profound shift in the proportion of pDC (46.0%) (Fig. 3B).

### Stimulation-specific genes in PBMCs exhibited varying effects in pig immunity

**Fig. 4:**
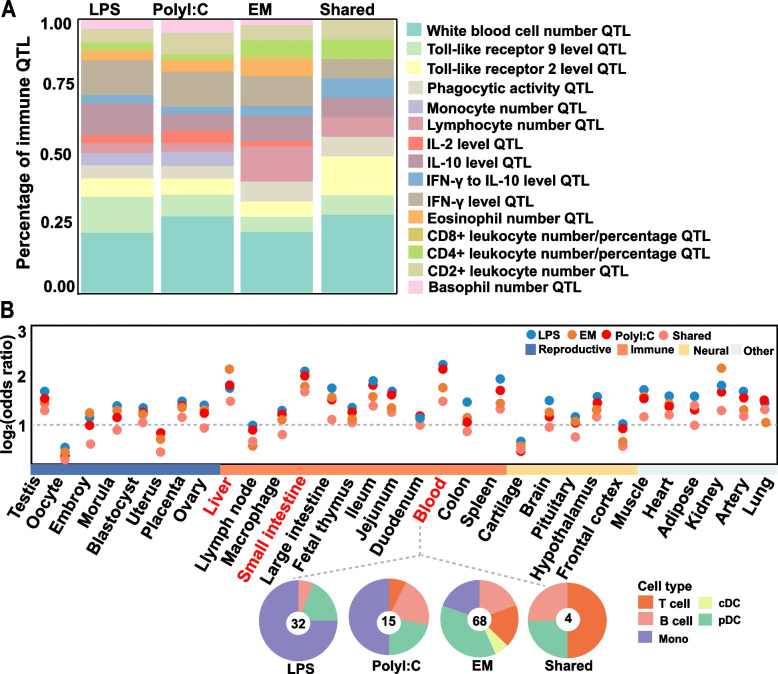
QTL annotation results for different stimulated specific DEGs as well as shared genes (**A**) and results of enrichment analysis with eGene from 31 tissues in the pigGTEX database (**B**), odds ratios were obtained using Fisher's exact test. Four scale plots showing the proportion of pig immune cell subpopulation-specific eGenes with different stimulations

Our results demonstrated that most DEGs in porcine PBMCs under different stimulations were stimulus-specific and showed pathogen-specific and cell-specific patterns. Subsequently, we explored whether these stimulus-specific genes were associated with the complex traits of pigs. Overall, 38.0% of the LPS-stimulated stimulus-specific genes and 25.6% of the PolyI:C-stimulated genes, respectively, were located in QTL regions associated with immune traits in pigs. However, only 4.8% of EM stimulation-specific genes were located in this region (Fig. 4A). Similarly, the results showed that white blood cell number QTL was the highest among all stimulations (34.3–41.2%), followed by IFN-γ level QTL (17.1–22.4%) (Fig. 4A). Furthermore, our results also showed that LPS- and PolyI:C-stimulated specific DEGs were enriched in QTLs associated with monocyte number QTL and phagocytic activity QTL, whereas the enrichment in CD4 + /CD8+ leukocyte number/percentage QTL was less pronounced.

To investigate the effect of genetic variation on the level of gene transcription induced by LPS and PolyI:C stimulation, we further integrated the specific genes of different stimulations and the eGene information of 31 tissues from the PigGTEX database (https://piggtex.farmgtex.org/) to obtain stimulation-specific eGenes. Enrichment analysis showed that the specific genes of LPS and PolyI:C stimulation were more enriched in immune tissues compared to other tissues, with the highest enrichment observed in the blood and small intestine (Fig. 4B). In addition, it was also found that genes of EM stimulation were enriched in higher fold with eGenes from the spleen and kidney than with LPS and PolyI:C stimulation (Fig. 4B).

### Pathogen-specific genetic variation regulates gene expression through cell-specific

**Fig. 5:**
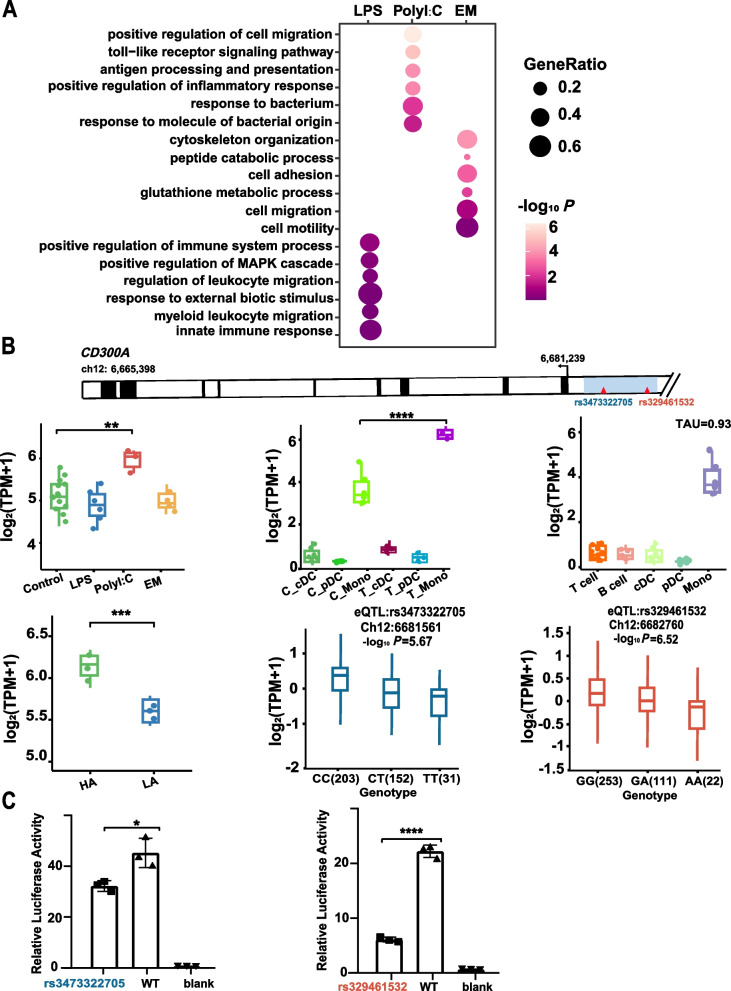
Luciferase assay of recombinant plasmid for key stimulus-specific genes in HEK293 cells. **A** GO terms for LPS-, EM-, and PolyI:C stimulus-specific eGene. **B** *CD300A*, a monocyte-specific gene, was significantly upregulated after Poly I:C stimulation in PBMCs and monocytes, and the promoter region was regulated by two SNPs. HA: high antibody level; LA: low antibody level.** C** Luciferase assay of the recombinant plasmids in HEK293 cells. Blank: Blank cells. PGL4.14: Empty vector. rs329461532: Plasmids of g.6682760C > T, rs3473322705: Plasmids of g.6681561G > A

eGenes serve as a vital linkage between genetic variation and the intricate regulation of gene expression [31]. Consequently, in our quest to pinpoint the crucial SNPs that govern gene expression in blood under specific stimuli, we utilized 6,076 blood-derived eGenes from the PigGTEX database [32]. This comprehensive analysis led us to identify a total of 232, 343, and 102 eGenes that are specifically responsive to LPS, EM, and PolyI:C stimuli, respectively, which are mainly associated with immune activation processes, including the innate immune response, leukocyte migration, and cell adhesion signaling pathways (Fig. 5A). Similar to previous findings in cattle and humans [33, 34], LPS- and PolyI:C-specific eGenes are mainly monocyte-specific (Fig. 4B). Moreover, we found that EM-specific eGenes were predominantly pDC-specific, and the genes shared across different stimuli included T-cell, pDC, and B-cell-specific genes. In addition, approximately 2.9%, 0.6%, and 1.9% of pDCs, cDCs, and mono-specific genes, respectively, responded positively to the PolyI: C-induced immune response after PolyI:C stimulation (Fig. S6B).

*CD300A* was specifically expressed in monocyte (TAU = 0.93), and its expression was significantly upregulated in both PBMCs and monocyte following viral mimic PolyI:C stimulation. *CD300A* is involved in the NF-κβ signaling pathway to activate host immunity and is also regulated by two eQTLs in the promoter (rs3473322705 and rs329461532) (Fig. 5B). In addition, porcine reproductive and respiratory syndrome virus (PRRSV) is also a class of RNA viruses that cause respiratory disease in pigs of all ages and reproductive disorders in sows. Next, we took a step to explore whether PRRSV stimulation induced the same expression profile of *CD300A*. The results showed that the expression of *CD300A* in PRRSV-stimulated piglets in the high-antibody (HA) group was significantly higher than that in the low-antibody (LA) group (Fig. 5B). Another example is *CD93*, which was identified as a marker gene for pDC (TAU = 0.90), and whose expression level was significantly upregulated in response to PolyI:C stimulation of PBMCs and pDC, regulated by an eQTL (rs1109431654) located in the promoter region (Fig. S7A).

Finally, we used a luciferase assay (Fig. 5C) to confirm these predictions. The luciferase activities of the T allele of rs3473322705 (g.6682760C > T) and allele A of rs329461532 (g.6681561G > A) were significantly higher than those of the blank control (*P* < 0.0001) and lower than those of allele C (*P* < 0.0001) and allele G (*P* = 0.0213) (Fig. 5C), respectively. These results indicate that the T allele of rs3473322705 and allele A of rs329461532 decreased the transcriptional activity of *CD300A* compared to alleles C and G. Similar results were confirmed for *CD93* (Fig. S7B). Taken together, our results suggest that environmental and genetic variants synergistically regulate gene expression and that certain genetic variants may act through cell specificity in response to specific stimuli.

### Context-specific genes were significantly enriched for GWAS signals of human immune and metabolic diseases

To investigate whether specific stimulated genes were enriched in GWAS signals characteristic of immunity or disease, we carried out GWAS enrichment analyses. However, the lack of publicly available GWAS data for pigs limited the enrichment of stimulus-specific genes with complex traits. Numerous studies have demonstrated that homologous genes are exhibit highly functional similarity. To further investigate whether pig stimulus-specific genes have similar expression profiles and functions in humans, we identified human cell-specific genes using RNA-Seq data from five human immune cell types (i.e., T-cells, B-cells, cDCs, pDCs, and mono). The analysis showed that the TAU values of cell-specific genes were highly correlated between pigs and humans (*P* < 0.0001) (Fig. S8, right panel). Moreover, the expression levels of the different major immune cell subpopulations in pigs were significantly correlated with the expression levels of their corresponding human immune cell homologs (*P* < 0.0001) (Fig. S8, left panel). These results indicate that the functional and expression profiles of porcine and human immune cells were highly similar, demonstrating the feasibility of human GWAS signal enrichment analysis.

**Fig. 6:**
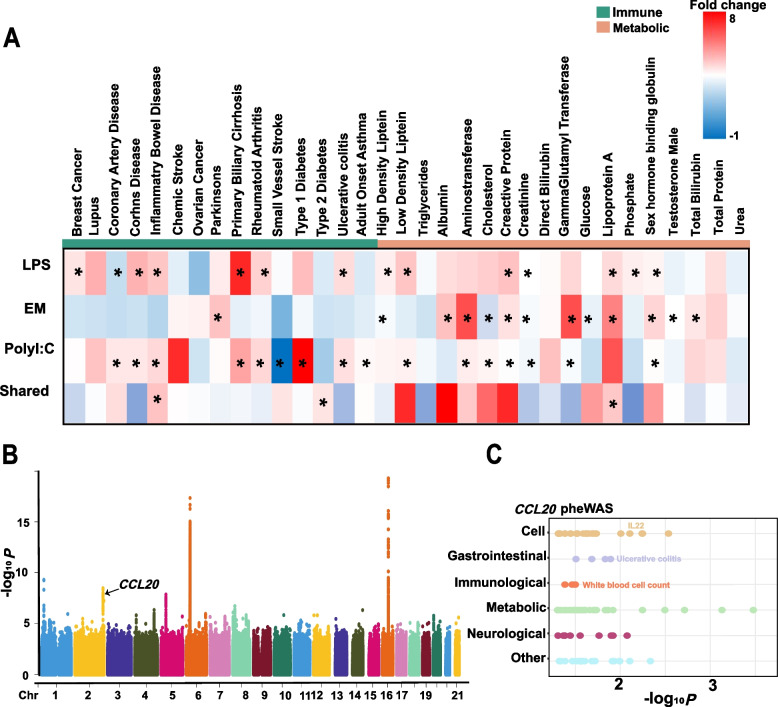
Results of enrichment analysis of stimulus-specific genes with GWAS signals for human complex traits and PheWAS analysis. **A** Results of enrichment analysis of stimulus-specific genes with GWAS signals for 33 complex traits of humans, “*” and indicate *P* < 0.05. **B** Manhattan plot of human inflammatory bowel disease GWAS, where the significance threshold is a *P* < 5e-08, and arrows indicate key genes identified. **C** Results of PheWAS analysis of *CCL20*

Thus, to explore the role of the identified pig stimulus-specific genes in resolving the mechanism of inheritance of human complex traits, we used data from 33 GWAS associated with human complex traits and performed a GWAS signal enrichment analysis for all stimulus-specific genes. The results revealed that pig-specific stimulated genes were enriched in GWAS signals for similar complex traits in humans. For example, LPS- and PolyI:C- specific expressed genes were significantly enriched for GWAS signals associated with inflammatory and immune-related diseases in humans (e.g., Crohn's disease, inflammatory bowel disease, and ulcerative colitis), whereas EM-specific expressed genes were predominantly enriched for metabolism-related GWAS signals, such as lipoprotein A and gamma-glutamyl transferase, Shared genes were significantly enriched in GWAS signaling with inflammatory bowel disease, type 2 diabetes, and lipoproteinA (Fig. 6A). *CCL20*, located on chromosome 2, is significantly enriched in GWAS signaling in inflammatory bowel disease and was identified as a gene specifically expressed in human and porcine monocyte (TAU_human = 0.94, TAU_pig = 0.87) (Fig. 6B, Supplemental Table S4). These results suggest that stimulation-specific genes have pleiotropic effects on many health and metabolic traits as well as on metabolic regulation in mammals, between humans and pigs. We then performed the PheWAS analysis of human direct homologs of these candidate genes in 3,302 human phenotypes (https://atlas.ctglab.nl/). The results showed that *CCL20* was also significantly correlated (*P* < 0.05) with immune (e.g., white blood cell count) and other health (metabolic and neurological) traits in humans (Fig. 6C).

Taken together, these results suggest that bacterial and viral stimuli induce transcriptional changes in pig immune cell subsets that are genetically linked to inflammatory and immune diseases in humans. These results suggest that pig pathogen-specific genes may provide novel insights into the molecular mechanisms underlying complex human metabolic and immune traits.

## Discussion

Tissue-specific and pathogen-specific regulation of gene expression has been extensively investigated in humans [35, 36]. However, little is known about the dynamic changes of gene expression patterns and cellular composition of pig PBMCs in response to different stimulations. Here, we analyzed RNA-seq data from LPS, PolyI:C stimulation, and exposure to various unknown microbial environments, providing a comprehensive understanding of the gene expression characteristics induced by bacterial, viral, and multiple pathogenic and microbial stimuli.

Gene expression showed strong pathogen-specific characteristics, and understanding the gene expression profiles induced by different stimuli and associated signaling pathways is crucial for resolving the host immune response mechanism. Differential expression and co-expression module analyses showed that the regulatory mechanisms of host immune response induced by different pathogen stimuli were different. LPS stimulation significantly activated NF-κβ and TNF signaling pathways, which was similar to the immune response induced by porcine PBMC to *actinobacillus pleuropneumoniae* infection [10]. Moreover, we discovered that similar to previous study on the gene expression signature of porcine PRRSV infection [8], PolyI:C stimulation significantly activated signaling pathways associated with antibody presentation and T-cell differentiation. Meanwhile, we found that invasion by different pathogens triggered alterations in the expression of certain genes associated with the immune response. LPS stimulation induced activation of NF-κβ target genes, such as cytokines, *IL19*, *IL1B2*, and *IL6ST* and chemokines *CCL8 and CCL21*, which is consistent with the findings of Lawlor et al. [37]. In addition, we found that PolyI:C stimulation induced the differential expression of pig leukocyte antigen genes, such as *SLA-DMB*, *SLA-DMA*, *SLA-DRA*, and *SLA-DQA1*. Numerous studies have shown that the structure and function of SLA genes are closely related to resistance and susceptibility to diarrhea [23], foot-and-mouth disease [38], and pig reproductive and respiratory syndrome [39], and that glycoproteins encoded by these genes on the cell surface play important roles in antigen presentation, immune response, and regulation [40]. In addition, our results revealed that bacterial and viral stimulation-specific differential genes were adjacent to monocyte and phagocytic capacity QTL at genomic locations. These data imply that bacterial and viral stimulation induces a dramatic innate immune response and that monocytes and phagocytosis play a key role in this process.

It has been well documented that the cellular composition of PBMCs varies significantly depending on the disease state and pathogenic stimulation [41]. Advances in single-cell technology can help identify the heterogeneity of cell subpopulations with specific functions and define the responses of cell subpopulations to validation and injury [42, 43]. Our study extends previous work by applying a method similar to single-cell analysis to quantify the proportion of the five major immune cells in PBMCs with different stimulations for the first time. Surprisingly, we found that the proportion of immune cells in PBMCs varied according to the type of stimulation, with monocytes being the core immune cells against viruses and bacteria. Our results are consistent with the findings of Gao et al. [33], where single-cell sequencing analysis of PBMCs demonstrated that LPS stimulation induced two activation states of CD14 + monocytes in PBMCs as well as upregulation of inflammation-related genes. Monocytes are the most important components of innate immunity, acting as antigen-presenting cells and mediating processes, such as T-cell activation and cytophagy. Our findings emphasize the central role of monocytes in the host immune response. However, we only used five major immune cell types in PBMCs to characterize the immune changes induced by different stimuli in PBMCs and lacked rare cell types, such as NK cells and macrophages. Furthermore, our study detected a substantial proportion of pDC cells in PBMC samples exposed to various unknown pathogens, exhibiting a pronounced enrichment of metabolic pathways, indicating a possible metabolic reprogramming (glycolytic) in support of their immune response [44]. However, the heterogeneity of the EM group environment compared to other stimuli necessitates caution in interpreting the average signal across cell populations. Therefore, future experiments are vital to validate the role of dendritic cells in mixed environmental stimuli. Next, we will integrate more cell types and single-cell data to validate our results. Furthermore, coupling transcriptional profiles to genetic variants allows the direct identification of genomic regulators of gene expression [45]. In the present study, we found that genetic variation under different stimuli may regulate gene expression through cell specificity, with more than 30.2% of viral- and bacterial-specific genes being immune cell-specific. This finding aligns with the work of Peters et al. [46], who proposed that the activation of immune cells under varying stimulus conditions can augment their functional properties of immune cells by increasing the transcription levels of cell-specific genes.

Extensive research demonstrates that pig and human organs exhibit substantial structural and functional homology, attributed to analogous gene functions [47, 48]. Our study aligns with these findings, revealing significant correlations in TAU values as well as expression levels of stimulus- and cell-specific genes between pigs and their human homologs [35]. LPS-stimulated pig PBMCs exhibited a similar gene expression profile to that of humans, activating NF-κB, cytokine-cytokine receptor, and toll-like receptor pathways to enhance cytokine gene expression and release [49, 50]. Remarkably, we observed a substantial enrichment of LPS-stimulated and PolyI:C-stimulated specific genes that were enriched with human GWAS signals, such as inflammatory bowel disease and Crohn's disease, diseases often accompanied by reduced intestinal bacterial diversity [51]. Similarly, genes specific to exposure to a wide range of pathogenic microbial environments are significantly enriched in most metabolic diseases (e.g., lipoprotein A, creatinine, and glucose), which is consistent with the function of EM-stimulated specific genes. The strong correlation between stimulus-specific genes and immune and metabolic diseases in this study illustrates the conserved nature of the regulatory functions of stimulus-specific genes and supports the pig as the most appropriate animal model for studying human diseases.

While our study provides valuable insights, it is crucial to acknowledge its limitations. Firstly, integrating RNA-seq data from various studies poses challenges due to technical discrepancies in RNA extraction, library preparation, ribose depletion or polyA selection, and sequencing platforms. Statistical methods cannot fully mitigate these confounders, and there may be potential unknown factors that remain undetected. To mitigate this, future efforts should strive for consistency in confounder control and similar sample collection conditions. Secondly, biological differences in the PBMC populations across samples, such as cell counts, animal health status, and stress levels, can affect gene expression levels. Future studies must account for these variations to isolate biological effects from technical artifacts. The sample size for our study was determined based on data available online, and samples that did not meet the criteria were excluded. Therefore, a smaller sample size may have led us to detect only a portion of true DEGs, such as only 402 DEGs were found in the PolyI:C-stimulation compared to the control (*P* < 0.05, log_2_(fold change) > 1.5). However, it is worth noting that the false-positive rate may also be relatively low for small sample sizes (perhaps representing 70% of all DEGs). With sample sizes less than 12, edgeR or DESeq2 performs well [52, 53], and in this study, there was an 89% overlap between Limma and DESeq2 in differential gene identifications. Also, we used similar criteria for the identification of DEGs as in previous studies [54, 55], and these results suggest that the present study has a certain level of confidence in the identification of differential genes. Future studies should consider increasing the sample size (at least *n* ≥ 6) to improve the detection rate of differentially expressed genes and minimize potential errors [52]. We believe that using a limited number of samples can still provide meaningful insights [56, 57]. For example, similar to the findings of Jiang et al. [56], we found that differential genes in virally stimulated PBMC samples significantly activated antigen presentation, T-cell activation, and cytokine receptor-associated signaling pathways compared to bacterial-stimulated PBMC.

In summary, our results provide a characterization of the gene regulatory landscape associated with the immune responses of different immune cells to bacterial and viral stimulation, illustrating the involvement of genetic variation in the host immune response in a pathogen-specific and cell-specific manner, regulating the expression of key immune-regulated genes and highlighting the central role of monocytes in the host immune response. Our results provide data to support our understanding of the genetic basis of immune traits and genetic breeding improvement efforts for disease resistance traits in pigs.

## Conclusions

Collectively, our study revealed shared and specific regulatory features after the stimulation of PBMCs in pigs by different pathogens and identified key SNPs regulating immune traits, emphasizing the synergistic role of genetic variation and the environment in regulating gene expression. These foundings provide important functional information for the selection and breeding of pigs against resistant diseases. In addition, the strong correlation between stimulus-specific genes and immune and metabolic diseases suggests a conserved role for these genes in the regulation of immune inflammation and potential pleiotropic effects on many metabolic traits in mammals, providing a reference that pigs can be used as model animals for humans.

## Materials and methods

### Sample information

The study comprehensively analyzed 128 publicly accessible RNA-seq datasets retrieved from the NCBI's SRA database, focusing on samples primarily originating from two commercial pig breeds and human immune cells (Fig. S1, S3 A, B), Table S1 shows all the RNA-seq details. Initially, 30 datasets were procured to delineate the gene regulatory patterns underlying pig PBMCs' immune responses to various stimuli. These samples were predominantly sourced from Landrace pigs or Yorkshire, and PBMCs were isolated from whole blood via density gradient centrifugation. To elucidate bacterial stimulation effects, 12 RNA-seq datasets were download [14], with six Landrace pigs treated with 5% CO_2_ (10 μg/mL) LPS for 2 h at 37 °C to elicit an optimal inflammatory response, while six served as untreated controls. Following treatment, cells were collected for RNA extraction. Additionally, six RNA-seq datasets derived from Landrace pigs were downloaded for characterizing viral stimulus-induced transcriptional changes in PBMCs [18]. Here, the treatment group (*n* = 3) was exposed to 20ug/mL PolyI:C for 24 h, marking the peak of the inflammatory response, while the control group (*n* = 3) was maintained in standard medium for 24 h, followed by RNA extraction. To mimic the porcine survival environment characterized by exposure to a wide range of microorganisms, we obtained RNA-seq data from six Yorkshire that were exposed to a soil-based environment during their early growth stages (days 4 to day 20) [19]. Specifically, the treatment group (*n* = 6) was exposed to an unknown microbial environment during this period, while the control group (*n* = 6) was reared conventionally in farrowing crates under similar conditions. At 56 days of age, both groups underwent the same process of peripheral blood mononuclear cell (PBMC) isolation from whole blood for RNA extraction.

To elucidate the dynamics of porcine peripheral blood immune cells in response to various stimuli, we acquired RNA-seq datasets of four studied porcine immune cell types. Study 1 was part of the FANG consortium and contained RNA-seq data for T cells, B cells, and monocytes isolated from two healthy Yorkshire pigs (*n* = 16) [26]. Study 2 and Study 3 complemented the missing cell types, both derived from unstimulated Yorkshire pigs. Study 2 focused on RNA-seq data for T cells (*n* = 5) [27] and Study 3 focused on RNA-seq data for cDC1, cDC2, pDC (*n* = 9) [28]. Data from study 4 were used to validate the gene expression profiles of cellular subpopulations in response to specific immune stimuli, and consisted primarily of RNA-seq data for PolyI:C-stimulated (10ug/mL) and unstimulated (both stimulated for 2 h) pDC, cDC2, and monocytes (*n* = 18) [29].

Subsequently, to explore the potential conservation of peacock-specific gene expression profiles and functions in humans, we accessed RNA-seq data from five unstimulated human immune cell types (T cells, B cells, cDCs, pDCs, and monocytes), primarily isolated from 17 healthy Singaporean individuals [57].

In addition, the study generated six RNA-seq datasets from piglets stimulated by the porcine reproductive and respiratory syndrome virus (PRRSV). Specifically, 1 mL of PRRS-MLV vaccine was injected intramuscularly into piglets at 28 d of age, followed by a second vaccination 30 d after vaccination. The piglets were reared under the same standard indoor conditions according to their growth stage. Blood samples were collected 21 d after the second vaccination (79-d-old), and serum PRRSV antibody levels were measured using an ELISA kit (HerdCheck PRRS 79X, IDEXX Laboratories Inc., USA). Finally, three piglets with high antibody level (HA, S/P = 499.0 ± 111.1) and low antibody level (LA, S/P = 227.0 ± 542.79) were selected based on the antibody level. The selected animals were examined via transcriptome analysis using peripheral PBMCs at 79 d of age. Unvaccinated neonatal piglets (*n* = 4) served as the controls. The PRRSV-stimulated piglet group used by Yang et al. provides information on production traits [8].

### PBMC isolation, RNA extraction, and RNA sequencing

First, 5 mL of fresh porcine blood was withdrawn in an anticoagulant tube, the blood was diluted according to the manufacturer's protocol, the Ficoll separation solution was added to the separation solution, and centrifuged at 1,700 rpm (~ 485 g) for 15–20 min at room temperature (18–22 °C). Blood was stratified according to the density of its components, and the middle layer was used to obtain PBMCs. Subsequently, total RNA was extracted directly from each sample using TRIzol (Invitrogen Life). Total RNA from porcine PBMCs was then extracted directly from each sample using TRIzol (Invitrogen Life Technologies, Carlsbad, CA, USA). RNA quality and integrity were measured using an Agilent Bioanalyzer 2100 (Agilent Technologies, Santa Clara, CA, USA). RNA-seq libraries were constructed using qualified RNA. Finally, the cDNA libraries were bipartite sequenced using 100 bp on the Illumina Hiseq2500 platform (Novogene, Beijing, China).

### Workflow for raw RNA-seq data

Briefly, the adapter was trimmed, and poor-quality reads were discarded using Trimmomatic [58] (v 0.39). Clean reads were compared with the porcine reference genome on Ensemble (Sscrofa11.1, v105) using STAR [59] (v2.7.0). Subsequently, gene expression was calculated using FeatureCounts [60] (v1.5.2), and transcripts per million (TPM) was obtained using Stringtie [61] (v2.1.1).

In this study, Low-expression genes were removed (counts < 6 in 40% of the samples), and a total of 13,823 genes were retained for subsequent analysis. Subsequently, we tested the effects of sex and breed on gene expression using linear regression models as described below:

$$model <- lm(gene\_expression, \sim gender + breed, data = count\_TPM\; normalized )$$

We determined the effect of confounding factors on gene expression based on the coefficients of the variables (Estimate Std) and *P* values [62]. The results showed that the coefficients for sex and breed did not reach statistically significant levels (*P* < 0.05). We identified confounders, such as cell counts and animal status, as batch effects from various research efforts. Subsequently, we minimized the impact of batch effects using the ComBat-seq function in the SVA package (R package) [63].

Principal component analysis (PCA) was used to visualize differences in gene expression between samples. Differential gene expression analysis was performed using the Limma [64]. The screening criteria for differentially expressed genes (DEGs) between the control and treated groups were* P* < 0.05, and log_2_ (fold change) > 1.5.

### Function enrichment analysis

We performed Kyoto Encyclopedia of Genomes (KEGG) [65] and Gene Ontology (GO) [66] enrichment analyses of genes using the online website KOBAS 3.0 ( http://bioinfo.org/kobas/) [67], and used *P* < *0.05* as the threshold for pathways to be significantly enriched.

### Cell-specific gene identification

The tissue specificity index (TAU) was calculated to quantify the cellular specificity of gene expression [68]. Based on the PCA results, untreated cells were classified as T cells, B cells, cDCs, pDCs, and mono. For each gene, the TPM of the gene in each cell type and the TAU value were calculated. A TAU value close to 1 indicates that the gene has high tissue specificity, while a TAU value close to 0 indicates that the gene is widely expressed in all tissues at similar levels. In this study, genes with TAU values > 0.7 were selected tissue-specific genes. Subsequently, the proportion of DEGs and specific genes in each cell-specific gene group was calculated for different stimulations.

### Weighted correlation network analysis (WGCNA) identified key modules and hub genes

A co-expression network containing 13,823 genes was constructed using WGCNA [69], setting a correlation coefficient threshold of 0.85 and choosing β = 8 to ensure a scale-free network based on the calculated results. After converting the filtered expression matrix into a topological overlap matrix, a hierarchical clustering analysis was performed for each gene, setting the minimum number of genes to 35, and modules with shear heights < 0.25 were combined into one module, and different stimulations were used as trait matrices to calculate whether different modules were correlated with each other. Heatmaps and dendrograms were used to visualize the results. Candidate modules associated with different stimuli were screened based on a correlation analysis heatmap and hierarchical clustering tree. Subsequently, key genes were determined based on module membership (MM) > 0.8 and gene significance (GS) > 0.5.

### Annotation of quantitative trait loci (QTLs) for immune traits and eGene enrichment analysis of immune tissues

To identify QTLs around the genes, this study downloaded pig QTLs (downloaded on April 13, 2023) from the available AnimalQTL database [70] (https://www.animalgenome.org/cgi-bin/QTLdb/index) and retained and analyzed the QTL upstream and downstream of the 10 kb stimulus-specific genes. In addition, enrichment analysis with these specifically expressed genes was performed using eGenes from 31 immune tissues in the PigGTEX database (PigGTEx-Portal (farmgtex.org), download on July 5, 2023) [32]. Considering the high tissue specificity of eGenes (genes whose expression level is influenced by at least one independent expression quantitative trait locus (eQTL)) [31], all tissues were classified into four types, including immune, reproductive, neural, and other tissues. *P* and enrichment ratios (odds ratio) were obtained using Fisher's exact test (*P* < 0.05).

### Construction of recombinant plasmid, cell culture, and luciferase assay

In this study, six luciferase reporter gene fragments with XhoI and HindIII restriction sites in the promoter region were used (Fig. S2), which contained alleles C and G of g.30240780C > G, alleles G and A of g.6681561G > A, and alleles C and T of g.6682760C > T. These six fragments were tested at Hitrobio Biotechnology Co., Ltd. (Beijing, China) and cloned into the pGL4.14 luciferase assay vector (Promega, Madison, USA). Subsequently, the plasmids were purified using an endonuclease-free Plasmid DNA Mini Kit II (Omega Bio-tek, Norcross, Georgia, USA) and sequenced to confirm the integrity of each construct insert.

Human embryonic kidney-293 T cells were cultured in Dulbecco's modified Eagle's medium containing 8% heat-inactivated fetal bovine serum (Gibco, Life Technologies) and 1% penicillin–streptomycin mixture (double antibody) at 5% CO_2_ and 37 °C. Approximately 2 × 10^5^ cells per well were seeded in 24-well plates and transfected using Lipofectamine 3000 (Invitrogen, CA, USA). Each well was transfected with 500 ng of constructed plasmid DNA and 10 ng of pRL-TK (Kidney Fluorescent Plasmid), replaced with fresh medium after 6 h, and assayed 24 h later using a dual luciferase reporter assay system with a modulus microtiter plate multi-mode reader (Promega) to measure firefly and renin luciferase activity (Turner Biosystems, California, USA). The average statistical value of three replicates was calculated as normalized luciferase data (firefly/renilla).

### Enrichment analysis of pig treatment-specific genes with human genome-wide association studies (GWAS) signals

A hierarchical linkage disequilibrium (LD) score regression was performed to test the enrichment of human homozygotes for pig stimulus-specific genes with the heritability of different traits [71], relies on the fact that the χ2 association statistic for a given SNP includes the effects of all SNPs that it tags.

χ2 association statistic for SNP j includes the effects of all SNPs that in LD with SNP j. That is, the expected χ2 statistic of SNP j is

$$E[{\chi }_{\text{j}}^{2}]=N{\sum }_{C}\tau c(j,C)+N\alpha +1$$

Here, N is the sample size of GWAS, C is the category index, and ℓ(j,C) is the LD score of SNP j relative to category C, defined as ℓ(*j*,*C*) = ${\sum }_{\kappa \epsilon C}{r}_{jk}^{2}$, where ${r}_{jk}^{2}$ is the squared correlation between SNP j and k in the population. *τc* is the contribution of annotation c to the heritability of each SNP conditional on other annotations. *τc* quantifies the importance of category C. If *τc* is 0, it indicates that category C is significantly enriched. If *τc* > 0, it suggests that category C increases the heritability of each SNP. In this study, we used the obtained *P*-value, which reflects the one-sided test of τc = 0, as a measure of whether the stimulus-specific gene expression program is enriched with the determinants of SNP heritability for a given trait. To ensure comparability among different traits, we normalized the heritability of each SNP, ${\sum }_{j}\text{Var }\left({\beta }_{j}\right)$, using the total number of SNPs, M, that contribute to the calculation of this heritability.

The code for LD score regression was from https://github.com/bulik/ldsc. The LD score was calculated using data from the European population of the 1000 Genomes Project (https://data.broadinstitute.org/alkesgroup/LDSCORE/1000G_Phase3_plinkfiles.tgz) [72], which was obtained from https://data.broadinstitute.org/alkesgroup/LDSCORE/1000G_Phase3_frq.tgz to download the minor allele frequencies of the single nucleotide polymorphisms (SNPs) in this population. Subsequently, summary statistics for the 33 traits were downloaded from a publicly available database (https://data.broadinstitute.org/alkesgroup/sumstats_formatted/). In this study, we extended the upstream and downstream regions of the genes for these traits by 10 kb. finally, a total of 1,000 tests were performed, and *P* < 0.05 was used as the threshold for significant enrichment.

### Phenome-wide association studies (PheWAS) analysis

The PheWAS analysis of human immediate homologs of stimulus-specific genes in 3,302 human phenotypes (https://atlas.ctglab.nl/) was performed [73]. PheWAS is a method used to examine the association between all phenotypes at the phenome-wide level and a given SNP, mainly to compensate for the shortcomings of GWAS in uncovering gene pleiotropy at the genome-wide level.

### Supplementary Information

Supplementary Material 1.

Supplementary Material 2.

## Data Availability

All raw data analyzed in this study are publicly available for download without restrictions from SRA (https://www.ncbi.nlm.nih.gov/sra/), GEO (https://www.ncbi.nlm.nih.gov/geo/), and EMBL-EBI (https://www.ebi.ac.uk/). Details of RNA-seq can be found in Supplemental Table S1 (Additional file 2_revised).

## Abbreviations

PBMCs: Peripheral blood mononuclear cells
LPS: Lipopolysaccharide
PolyI:C: Polyinosinic acid
EM: Exposure to multiple pathogens and microbial
DEGs: Differentially expressed genes
WGCNA: Weighted correlation network analysis
TOM: Topological overlap matrix
GWAS: Genome-wide association studies
NCBI: National center for biotechnology information
SRA: Sequence read archive
cDC: Conventional dendritic cells
pDC: Plasmacytoid dendritic cells
Mono: Monocyte
QTL: Quantitative trait loci
KEGG: Kyoto encyclopedia of genes and genomes
GO: Gene ontology
PCA: Principal component analysis
TPM: Transcript per million
eQTL: Expression quantitative trait loci
Phe-WAS: Phenome-wide association studies
