# Supplementary material for: Pathogen stimulations and immune cells synergistically affect the gene expression profile characteristics of porcine peripheral blood mononuclear cells
Source: BMC Genomics. 2024 Jul 25;25:719. doi: 10.1186/s12864-024-10603-9 (PMC11270792; doi:10.1186/s12864-024-10603-9)
Supplement: Supplementary file 1 — Supplementary Material 1. [file 12864_2024_10603_MOESM1_ESM.docx]

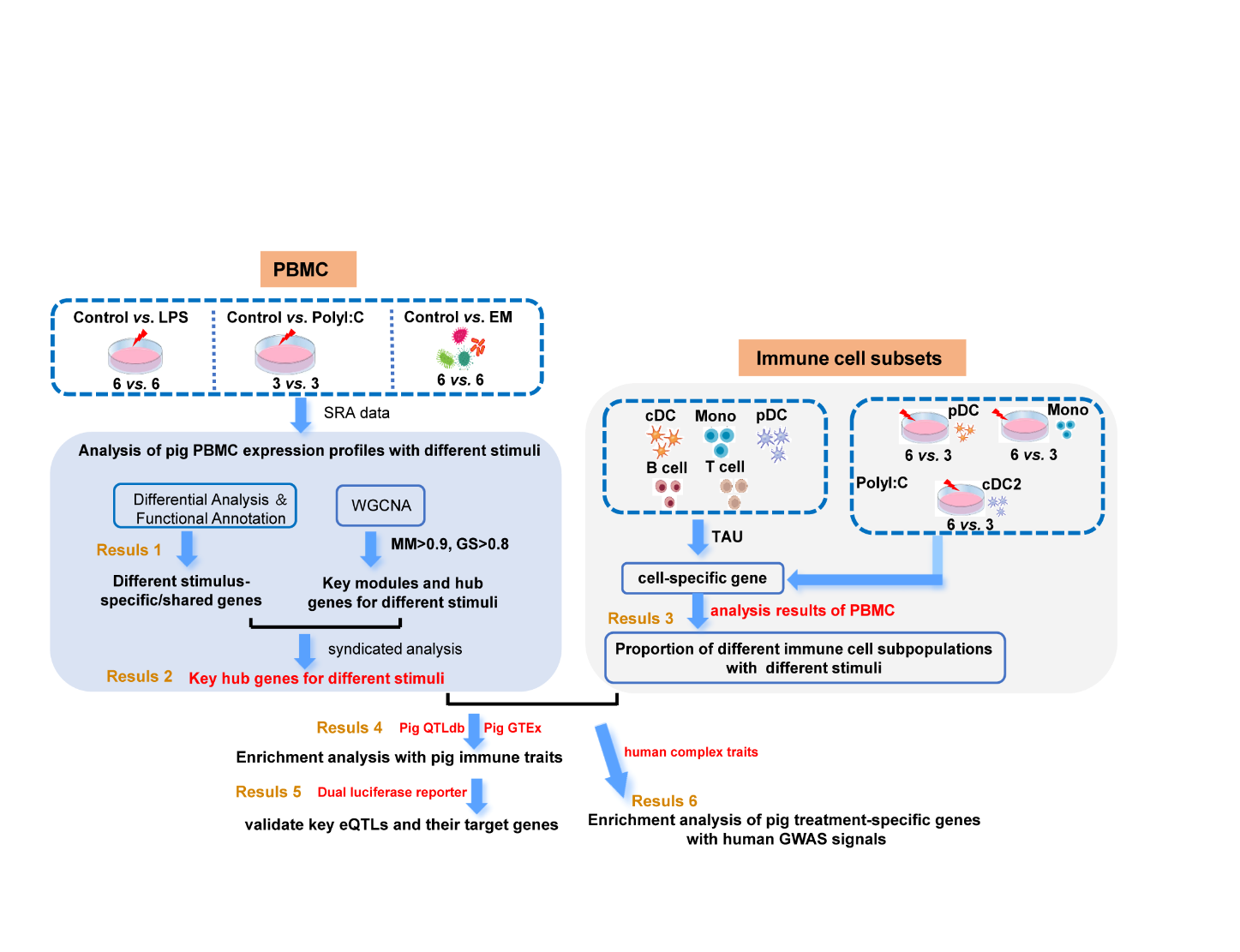


**Fig. S1** The overview design of this study. Using 128 RNA-seq data publicly available from the National Center for Biotechnology Information (NCBI) Sequence Repository (SRA) database, we systematically compared the transcriptional differences in pig PBMCs induced by different stimuli, including bacterial, viral, and a variety of unknown pathogens stimuli, and mined to obtain the key modules and hub genes using WGCNA and enrichment analysis. We also investigated the role of genetic variation in the regulation of gene expression under different stimuli and probed the dynamics of immune cells in PBMCs under various stimuli by integrating the results of a large-scale genome-wide association study (GWAS) of pig quantitative trait loci (QTLs), as well as expression quantitative trait loci (eQTLs) and 33 human complex traits.


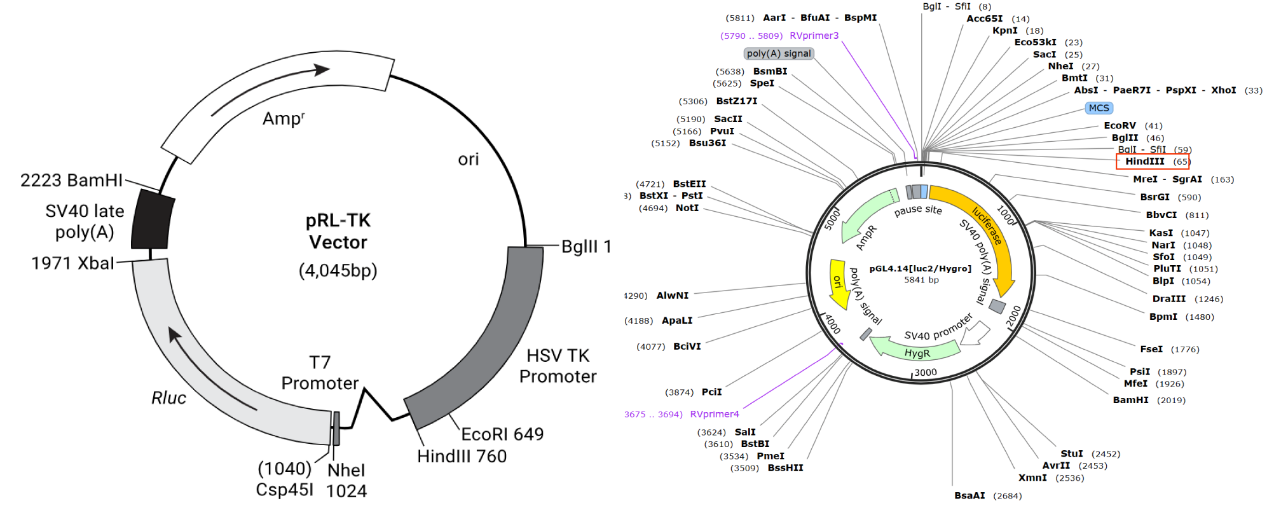


**Fig. S2** The diagram of luciferase reporters used in this study. The red boxes represent the restriction sites of *CD300A* and *CD93*. pRL-TK vector: a control reporter vector; PGL4.14 vector: identify fragment harboring the functional variant.


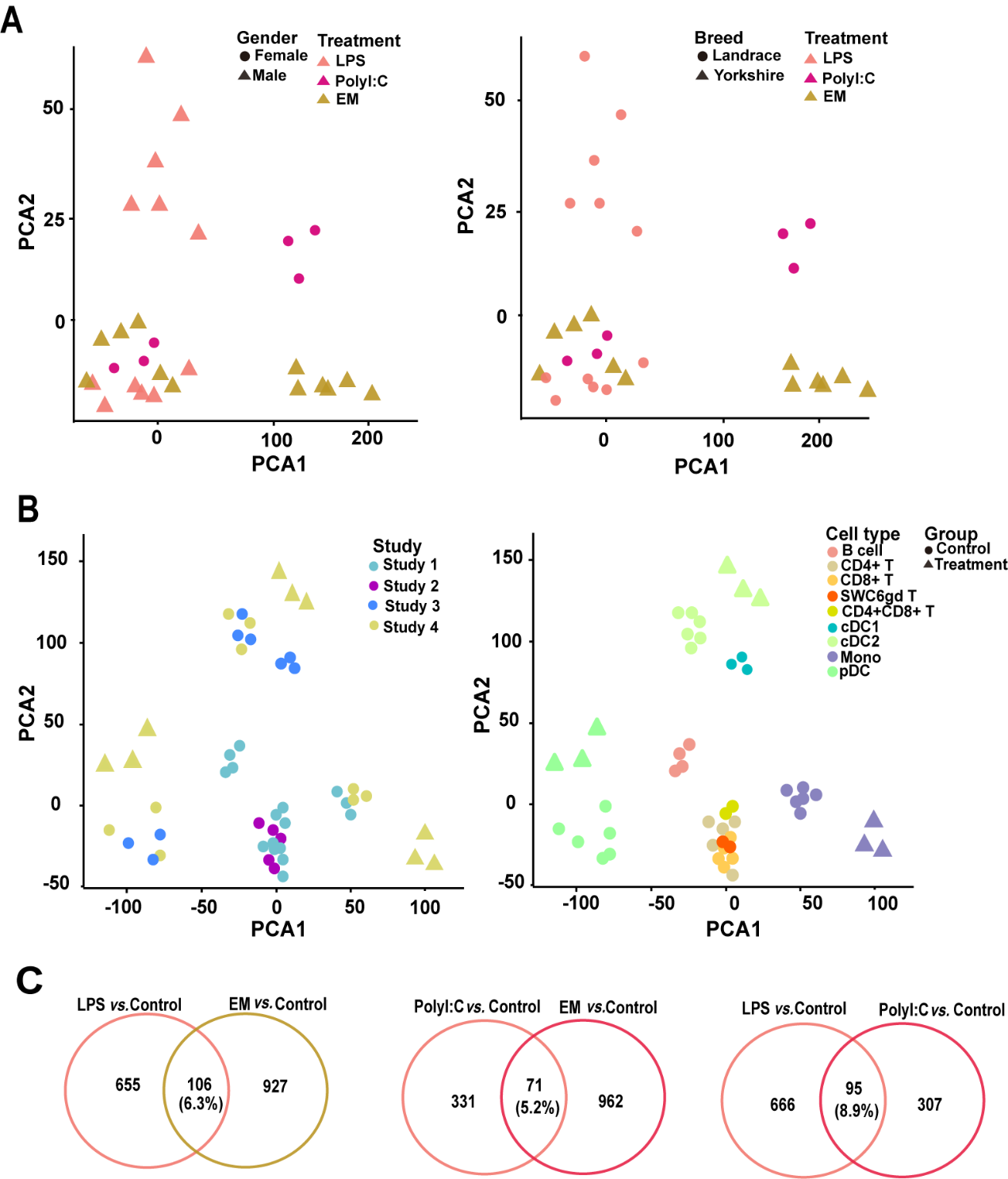


**Fig S3.** Differences in gene expression profiles of pig PBMCs and different immune cell subpopulations under different stimuli. **A** Principal Component Analysis (PCA) analysis of all genes after stimulation of PBMCs by LPS, PolyI:C, and various pathogens and microorganisms (EM). **B** The left panel shows the details of immune cell subsets data from four studies. Study 1 contains RNA-seq data for T cells, B cells, and monocytes isolated from two healthy Yorkshire pigs. Missing cell types were supplemented by Study 2 and Study 3, both from unstimulated Yorkshire pigs. Study 2 focused on RNA-seq data for T cells and Study 3 focused on RNA-seq data for cDC1, cDC2, and pDC. Data from Study 4 were used to validate the gene expression profiles of cellular subpopulations in response to specific immune stimuli and consisted primarily of RNA-seq data from PolyI:C-stimulated and non-stimulated pDC, cDC2 and monocytes. **C** Differential genes shared between different stimuli.


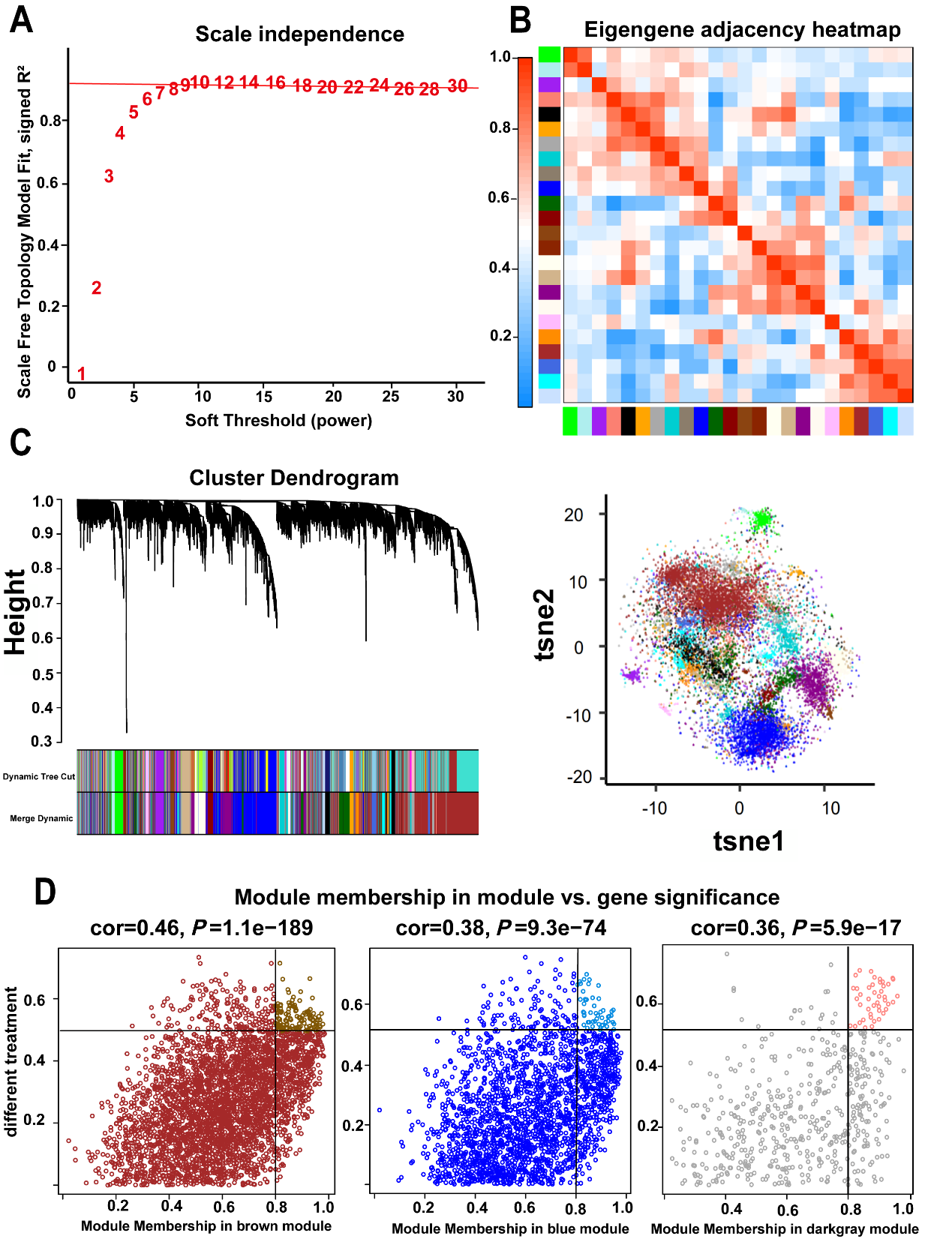


**Fig. S4** The weighted gene correlation network analysis (WGCNA) for 30 RNA-Seq datasets. **A** Analysis of the scale-free index for various soft-threshold powers (β). B Heatmap of correlation between modules. The horizontal and vertical axes represent different modules, the weaker the correlation, the blue color, the stronger the correlation, the red color. C Dendrogram of all differentially expressed genes clustered based on the measurement of dissimilarity (1-TOM) (left panel). The color band shows the results obtained from the automatic single-block analysis. Clustering map of different module genes (right panel). **D** Scatterplot of gene significance (GS) for stimulus vs. module membership (MM) in candidate modules. The figure shows the significance of the genes in the three modules. The x-axis represents the value of membership in each module. The three plots represent genes in the brown, blue, and dark gray modules, respectively. The gene in the upper right corner of each graph is the required hub gene.


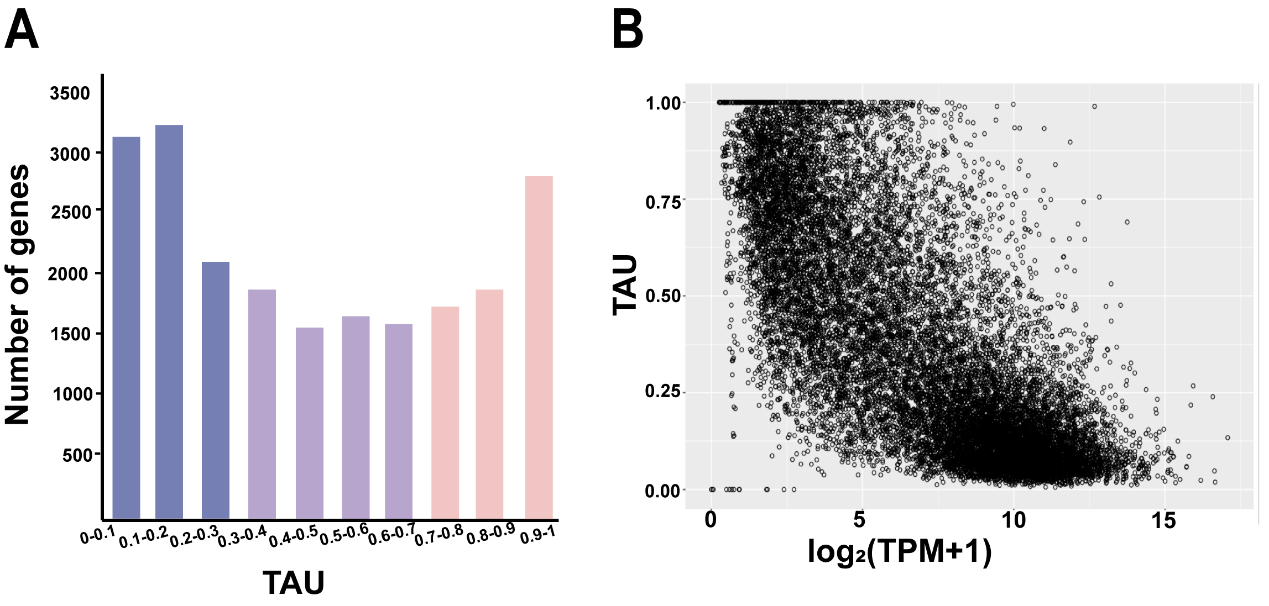


**Fig. S5** Identification of cell-specific genes for different immune cells. **A** Distribution of TAU values for all genes. We divided the TAU values into three intervals, with 0-0.3 representing genes shared between cells, 0.3–0.7 representing low-specificity genes, and 0.7-1 representing genes with cell-specificity **B** Correlation between TAU values and gene expression levels.


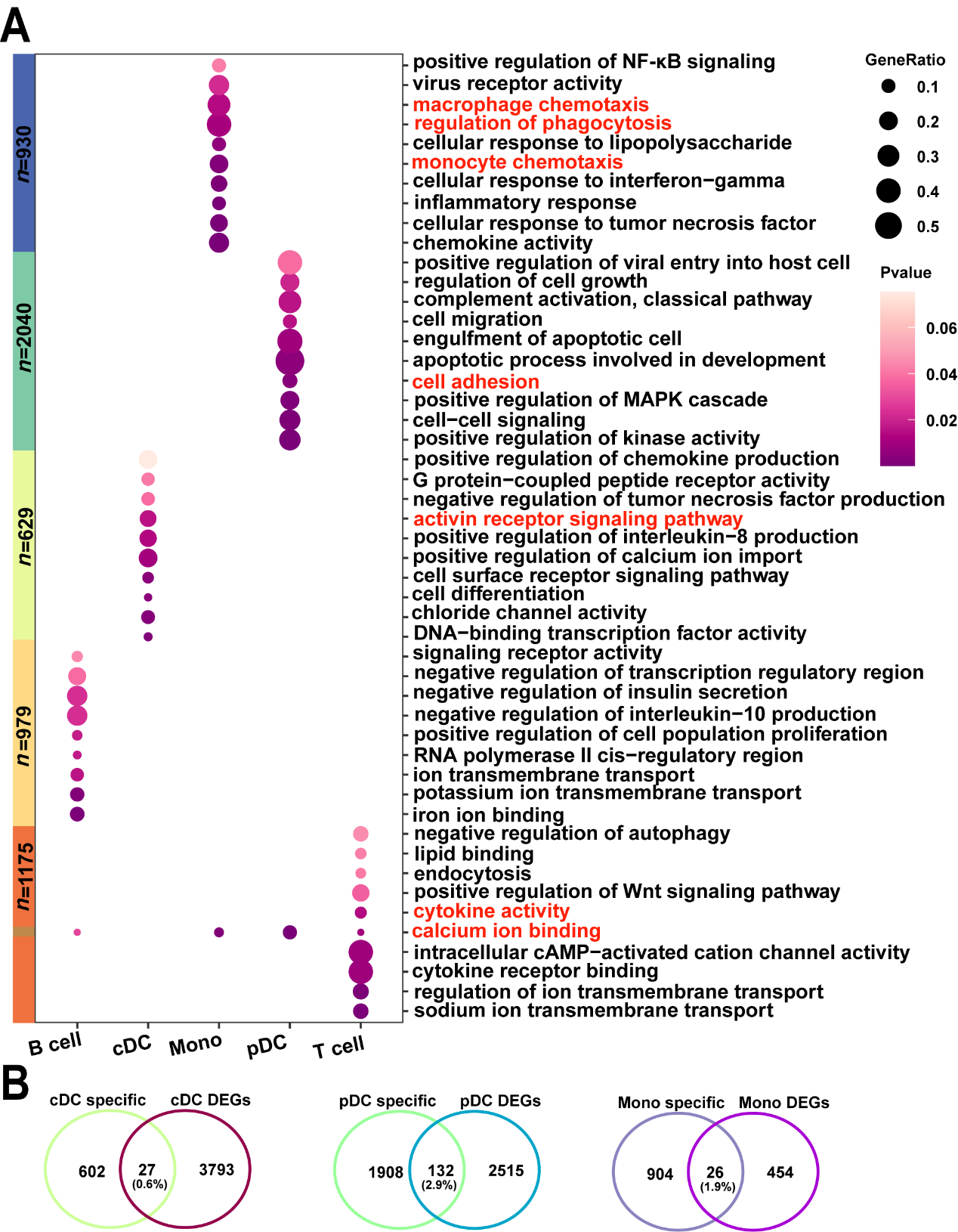


**Fig. S6** Functional enrichment analysis of cell-specific genes. **A** Results of GO analysis of specific genes of five immune cell subsets. **B** Genes shared between cell-specific genes and DEGs (*P* < 0.05 and log_2_ (fold change) >1.5) with PolyI:C stimulation of three immune cell subsets.


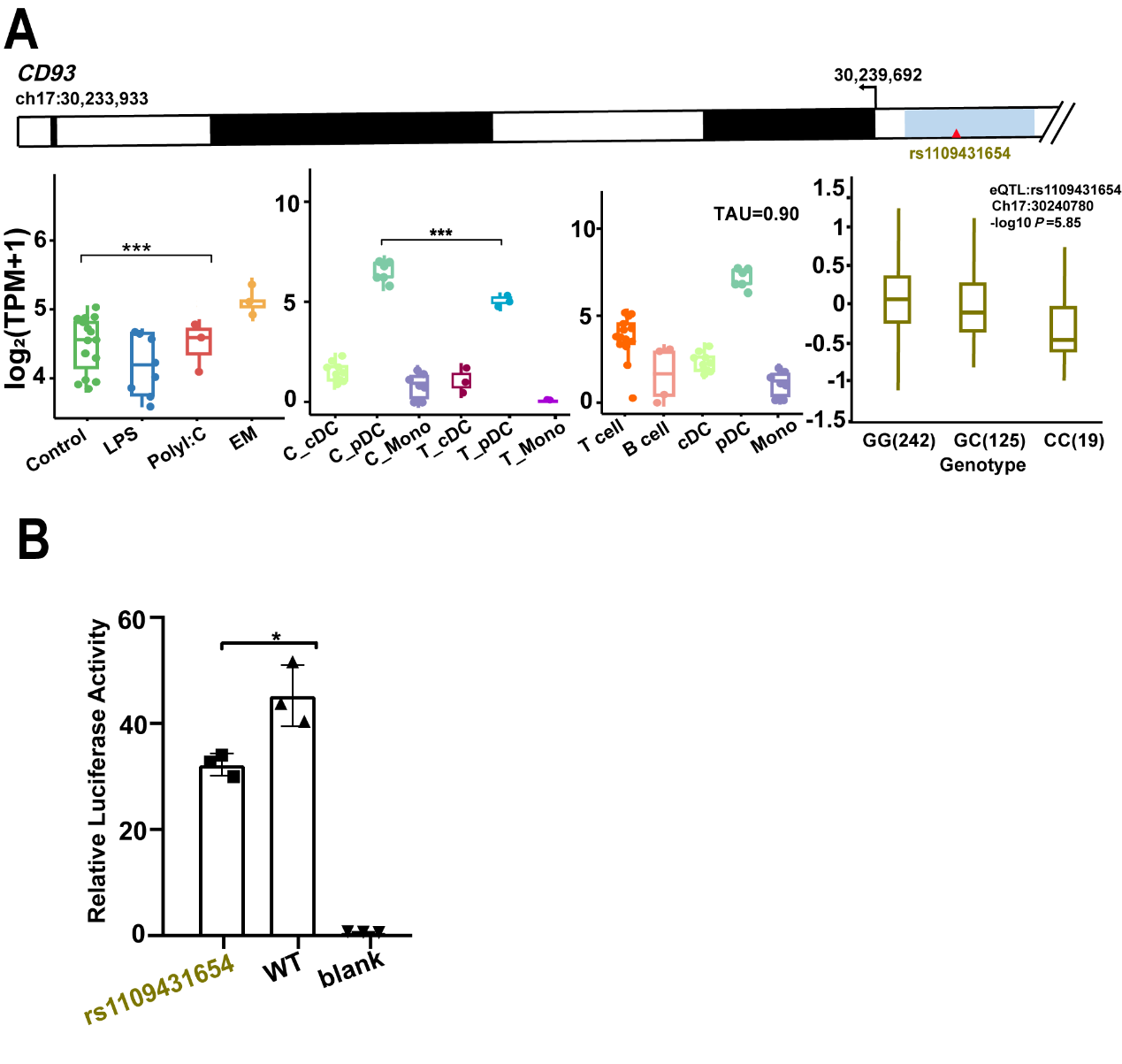


**Fig. S7** Luciferase assay of recombinant plasmids for key stimulus-specific genes in HEK293 cells.**A** The expression level of the pDC-specific gene *CD93* was significantly upregulated by PolyI:C stimulation in PBMC and pDCs, and its promoter region was regulated by one SNP. **B** Luciferase assay of the recombinant plasmids in HEK293 cells. Blank: blank cells. pgl4.14: empty vector. rs11094316541: Plasmid for g.30240780G>C.


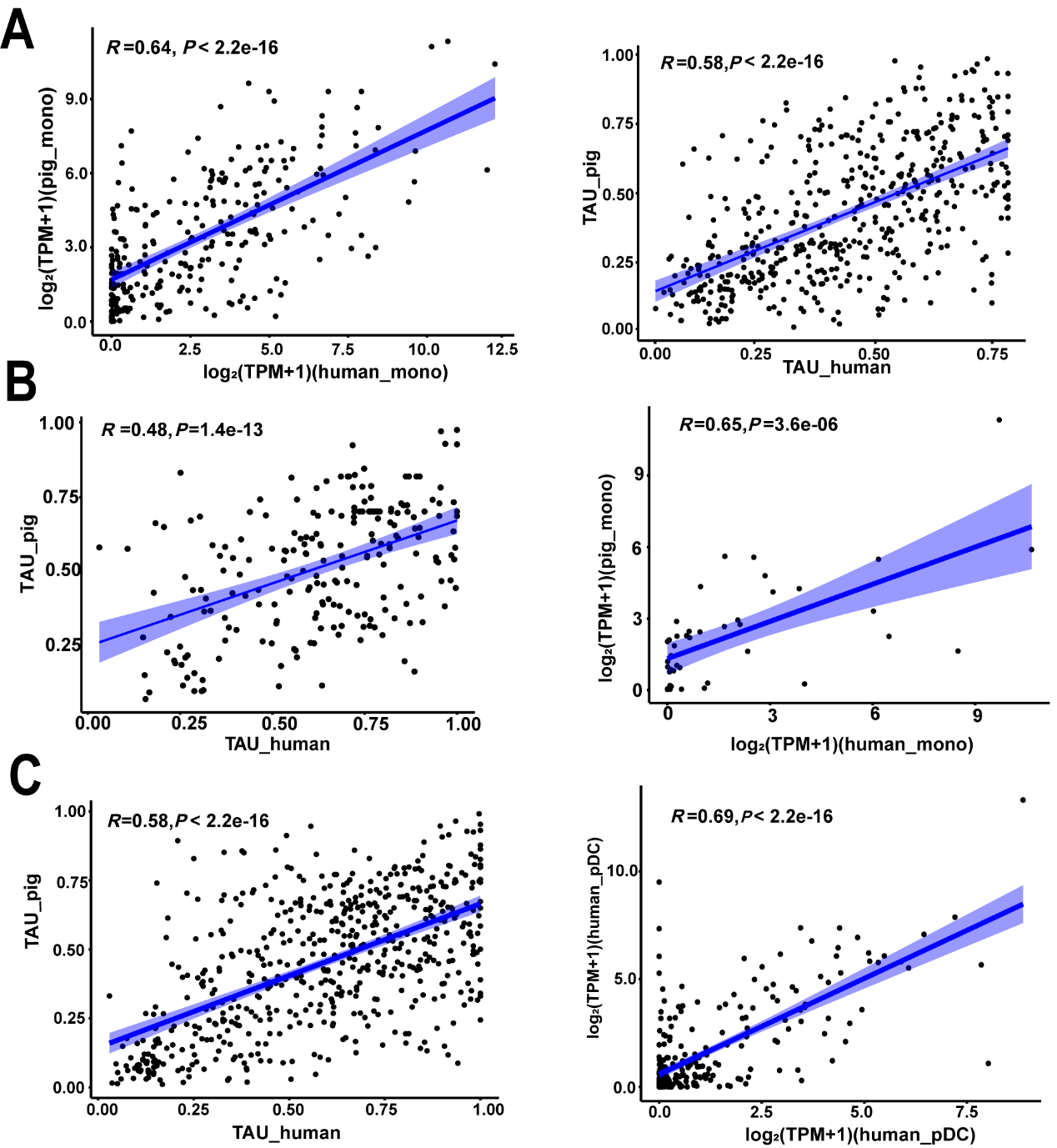


**Fig. S8** Correlation of different stimulus-specific genes with their human homologs in pigs. The left panels of **A**, **B**, and **C** show the correlation of TAU values of LPS, PolyI:C, and EM-stimulated specific genes with those of their human homologs, respectively, and the right panels show the correlation of expression levels of different stimulated genes with their homologs of human immune cell specific genes, with LPS and PolyI:C stimulation mainly in monocytes and EM stimulation mainly in pDCs.
