# Supplementary material for: Pathogen stimulations and immune cells synergistically affect the gene expression profile characteristics of porcine peripheral blood mononuclear cells
Source: BMC Genomics. 2024 Jul 25;25:719. doi: 10.1186/s12864-024-10603-9 (PMC11270792; doi:10.1186/s12864-024-10603-9)
Supplement: Supplementary file 2 — Supplementary Material 2. [file 12864_2024_10603_MOESM2_ESM.docx]

**Supplemental Table S1** The summary list of RNA-seq datasets used in this study

|  | **Study** **description** | **type** | **Information** | **Accession** | **Used for result** | **PBMC collection/DOI** |
| --- | --- | --- | --- | --- | --- | --- |
| LPS | Simulated with LPS (10 μg/mL) for 2h  mixture of cell culture medium and PBS for 2h | PBMC  (*n* = 12) | Landrace  male | EMBL-EBI,  E-MTAB-9808 | Result 1,2,3,4,5,6 | Electronarcosis and dissected; doi:10.1080/21505594.2021.1948276 |
| PolyI:C | Simulated with PolyI:C (20 μg/mL) for 24h  mixture of cell culture medium and PBS for 24h | PBMC  (*n* = 6) | Landrace  female | SRA,  PRJNA301538 | Result  1,2,3,4,5,6 | Venipuncture via jugular vena cava; doi:10.1534/g3.116.028290/-/DC1 |
| EM | exposed daily to fresh topsoil from (day 4 -d 20)  reared conventionally in farrowing crates | PBMC  (*n* = 12) | Yorkshire  male | GEO, GSE205321 | Result  1,2,3,4,5,6 | Venipuncture via jugular vena cava;  doi:10.3389/fgene.2022.886875 |
| PRRSV | PBMC stimulation in pigs using PRRSV-MLV | PBMC  (*n* = 6) | Landrace  male | SRA,  PRJNA390045 | Result 5 | Venipuncture via jugular vena cava;  doi: 10.1038/s41598-018-20701-w |
| \  \  \ | RNA-seq data for five immune cell subsets (T cell, B cell, Monocyte)  RNA-seq data for T cell  RNA-seq data for three immune cell subsets (cDC2, cDC1, pDC) | Immune cell  (*n* = 16)  Immune cell  (*n* = 5)  Immune cell  (*n* = 9) | Yorkshire  male  Yorkshire  male/female  Yorkshire  male | SRA,  PRJEB43826  SRA,  PRJEB27455  SRA,  PRJEB15381 | Result 3,4,5  Result 3,4,5  Result 3,4,5 | Venipuncture via jugular vena cava;  doi:10.1177/1753425920951607  /  doi： 10.1186/s12915-019-0726-5  /  doi:10.4049/jimmunol.1600672 |
| PolyI:C | Simulated with PolyI:C (10 μg/mL) for 2h (pDCs, cDC2, and Monocyte) | Immune cell  (*n* = 18) | Yorkshire  female | SRA,  PRJEB37564 | Result 3,4,5 | /  doi:10.3389/fimmu.2020.01429 |
| \ | five human immune cell subsets (T cell, B cell, pDCs, cDCs, Monocyte) | Immune cell  (*n* = 44) | Singaporean female/male | GEO,  GSE107011 | Result 6 | /  doi:10.1016/j.celrep.2019.01.041 |

**Supplemental Table S2** KEGG results of different stimulations

| **Group** | **Term** | **number** | **-log10(*P*-Value)** | **Input** |
| --- | --- | --- | --- | --- |
| **LPS** | Cytokine-cytokine receptor interaction | 21 | 7.65 | ENSSSCG00000016254\|ENSSSCG00000017044  \|ENSSSCG00000011730\|ENSSSCG00000008953 |
|  | NF-kappa B signaling pathway | 13 | 7.26 | ENSSSCG00000013655\|ENSSSCG00000008953\|  ENSSSCG00000010312\|ENSSSCG00000001952 |
|  | TNF signaling pathway | 13 | 6.57 | ENSSSCG00000016254\|ENSSSCG00000027426\|  ENSSSCG00000013655\|ENSSSCG00000014277 |
|  | Toll-like receptor signaling pathway | 9 | 3.98 | ENSSSCG00000017700\|ENSSSCG00000011899  \|ENSSSCG00000008953\|ENSSSCG00000017044 |
|  | C-type lectin receptor signaling pathway | 9 | 3.69 | ENSSSCG00000027426\|ENSSSCG00000011730\|  ENSSSCG00000014277\|ENSSSCG00000017044 |
|  | IL-17 signaling pathway | 8 | 3.56 | ENSSSCG00000016254\|ENSSSCG00000008953  \|ENSSSCG00000004420\|ENSSSCG00000012967 |
|  | NOD-like receptor signaling | 9 | 2.88 | ENSSSCG00000008953\|ENSSSCG00000015435\|  ENSSSCG00000012853\|ENSSSCG00000001952 |
|  | Th1 and Th2 cell differentiation | 7 | 2.74 | ENSSSCG00000011730\|ENSSSCG00000001703\|  ENSSSCG00000017044\|ENSSSCG00000004755 |
|  | MAPK signaling pathway | 13 | 2.66 | ENSSSCG00000008881\|ENSSSCG00000010448\|  ENSSSCG00000014441\|ENSSSCG00000003573 |
| **EM** | Metabolic pathways | 39 | 2.46 | ENSSSCG00000003922\|ENSSSCG00000013046  \|ENSSSCG00000010742\|ENSSSCG00000025942 |
|  | Cell adhesion molecules (CAMs) | 9 | 2.37 | ENSSSCG00000006288\|ENSSSCG00000029267  \|ENSSSCG00000008217\|ENSSSCG00000008601 |
|  | Autophagy - other | 4 | 1.96 | ENSSSCG00000002707\|ENSSSCG00000000645\|  ENSSSCG00000015842\|ENSSSCG00000012569 |
|  | Tight junction | 9 | 1.68 | ENSSSCG00000011497\|ENSSSCG00000012071  \|ENSSSCG00000005247\|ENSSSCG00000006564 |
|  | Histidine metabolism | 3 | 1.55 | ENSSSCG00000004644\|ENSSSCG00000009889  \|ENSSSCG00000016442 |
|  | PPAR signaling pathway | 5 | 1.53 | ENSSSCG00000001844\|ENSSSCG00000016199  \|ENSSSCG00000009245\|ENSSSCG00000014272 |
|  | Cholesterol metabolism | 4 | 1.49 | ENSSSCG00000025020\|ENSSSCG00000016199  \|ENSSSCG00000006831\|ENSSSCG00000015405 |
|  | Staphylococcus aureus infection | 4 | 1.47 | ENSSSCG00000020934\|ENSSSCG00000007797  \|ENSSSCG00000006288\|ENSSSCG00000023868 |
|  | Oxidative phosphorylation | 7 | 1.33 | ENSSSCG00000024550\|ENSSSCG00000013046  \|ENSSSCG00000007524\|ENSSSCG00000011893 |
| **PolyI:C** | Antigen processing and presentation | 7 | 6.42 | ENSSSCG00000001453\|ENSSSCG00000001469\|  ENSSSCG00000001456\|ENSSSCG00000001457 |
|  | Phagosome | 10 | 6.42 | ENSSSCG00000011848\|ENSSSCG00000006979\|  ENSSSCG00000001453\|ENSSSCG00000001469 |
|  | Th17 cell differentiation | 8 | 5.61 | ENSSSCG00000001453\|ENSSSCG00000001469  \|ENSSSCG00000001456\|ENSSSCG00000001457 |
|  | Th1 and Th2 cell differentiation | 7 | 5.03 | ENSSSCG00000001453\|ENSSSCG00000001469\|  ENSSSCG00000001456\|ENSSSCG00000001457 |
|  | Viral protein interaction with cytokine and cytokine receptor | 4 | 2.24 | ENSSSCG00000023852\|ENSSSCG00000016322  \|ENSSSCG00000015653\|ENSSSCG00000016916 |
|  | Leukocyte transendothelial migration | 4 | 1.93 | ENSSSCG00000002829\|ENSSSCG00000007718\|  ENSSSCG00000005743\|ENSSSCG00000015959 |
|  | IL-17 signaling pathway | 3 | 1.71 | ENSSSCG00000023852\|ENSSSCG00000023737  \|ENSSSCG00000014988 |
|  | Cytokine-cytokine receptor interaction | 6 | 1.63 | ENSSSCG00000015653\|ENSSSCG00000002385  \|ENSSSCG00000023852\|ENSSSCG00000016322 |
|  | Natural killer cell mediated cytotoxicity | 3 | 1.36 | ENSSSCG00000006374\|ENSSSCG00000023737  \|ENSSSCG00000005743 |
| **Shared** | Jak-STAT signaling pathway | 2 | 2.27 | ENSSSCG00000012699\|ENSSSCG00000015652 |
|  | Chemokine signaling pathway | 2 | 2.18 | ENSSSCG00000006298\|ENSSSCG00000015324 |
|  | PI3K-Akt signaling pathway | 2 | 1.99 | ENSSSCG00000015324\|ENSSSCG00000013501 |
|  | Cytokine-cytokine receptor interaction | 2 | 1.65 | ENSSSCG00000006298\|ENSSSCG00000015652 |

**Supplemental Table S3** GO terms of cell-specific genes

| **Group** | **Description** | **GeneRatio** | ***P* value** | **Input** |
| --- | --- | --- | --- | --- |
| **T cell** | sodium ion transmembrane transport | 0.13 | 0.000378 | ENSSSCG00000026527\|ENSSSCG00000017120\|  ENSSSCG00000006811\|ENSSSCG00000011577 |
|  | regulation of ion transmembrane transport | 0.14 | 0.000654 | ENSSSCG00000028112\|ENSSSCG00000026527\|  ENSSSCG00000000106\|ENSSSCG00000015913 |
|  | cytokine receptor binding | 0.40 | 0.008903 | ENSSSCG00000014281\|ENSSSCG00000009087 |
|  | intracellular cAMP-activated cation channel activity | 0.40 | 0.008903 | ENSSSCG00000006134\|ENSSSCG00000026527 |
|  | calcium ion binding | 0.04 | 0.009184 | ENSSSCG00000009203\|ENSSSCG00000017304\|  ENSSSCG00000017307\|ENSSSCG00000016416 |
|  | cytokine activity | 0.07 | 0.013951 | ENSSSCG00000014281\|ENSSSCG00000014282\|  ENSSSCG00000000363\|ENSSSCG00000009087 |
|  | positive regulation of Wnt signaling pathway | 0.17 | 0.034966 | ENSSSCG00000016416\|ENSSSCG00000002836 |
|  | endocytosis | 0.06 | 0.041017 | ENSSSCG00000009672\|ENSSSCG00000000854  \|ENSSSCG00000016416\|ENSSSCG00000004453 |
|  | lipid binding | 0.06 | 0.042528 | ENSSSCG00000006970\|ENSSSCG00000029295  \|ENSSSCG00000024787\|ENSSSCG00000026137 |
|  | negative regulation of autophagy | 0.13 | 0.045561 | ENSSSCG00000015879\|ENSSSCG00000025188 |
| **B cell** | iron ion binding | 0.12 | 0.00038 | ENSSSCG00000025273\|ENSSSCG00000016199\|  ENSSSCG00000029606\|ENSSSCG00000003754 |
|  | potassium ion transmembrane transport | 0.10 | 0.002208 | ENSSSCG00000003449\|ENSSSCG00000013031\|  ENSSSCG00000017005\|ENSSSCG00000002423 |
|  | ion transmembrane transport | 0.09 | 0.015823 | ENSSSCG00000009197\|ENSSSCG00000003640\|  ENSSSCG00000012129\|ENSSSCG00000004827 |
|  | RNA polymerase II cis-regulatory region sequence-specific DNA binding | 0.049 | 0.017438 | ENSSSCG00000010638\|ENSSSCG00000011618\|  ENSSSCG00000017539\|ENSSSCG00000005089 |
|  | positive regulation of cell population proliferation | 0.06 | 0.017929 | ENSSSCG00000016830\|ENSSSCG00000010698\|  ENSSSCG00000022833\|ENSSSCG00000016705 |
|  | negative regulation of interleukin-10 production | 0.25 | 0.023075 | ENSSSCG00000007007\|ENSSSCG00000017630 |
|  | negative regulation of insulin secretion | 0.25 | 0.023075 | ENSSSCG00000006002\|ENSSSCG00000025822 |
|  | calcium ion binding | 0.04 | 0.028399 | ENSSSCG00000014599\|ENSSSCG00000004678\|  ENSSSCG00000010926\|ENSSSCG00000024781 |
|  | negative regulation of transcription regulatory region DNA binding | 0.18 | 0.038137 | ENSSSCG00000004223\|ENSSSCG00000005586 |
|  | signaling receptor activity | 0.06 | 0.044979 | ENSSSCG00000009197\|ENSSSCG00000003640\|  ENSSSCG00000000614\|ENSSSCG00000001921 |
| **cDC** | DNA-binding transcription factor activity | 0.05 | 0.001536 | ENSSSCG00000008165\|ENSSSCG00000007710\|  ENSSSCG00000005236\|ENSSSCG00000005233 |
|  | chloride channel activity | 0.10 | 0.002926 | ENSSSCG00000025708\|ENSSSCG00000028922  \|ENSSSCG00000012755\|ENSSSCG00000021902 |
|  | cell differentiation | 0.04 | 0.003959 | ENSSSCG00000006056\|ENSSSCG00000013281\|  ENSSSCG00000005369\|ENSSSCG00000016635 |
|  | cell surface receptor signaling pathway | 0.07 | 0.004028 | ENSSSCG00000015320\|ENSSSCG00000006348\|  ENSSSCG00000013015\|ENSSSCG00000001589 |
|  | positive regulation of calcium ion import | 0.20 | 0.010142 | ENSSSCG00000009642\|ENSSSCG00000015895 |
|  | positive regulation of interleukin-8 production | 0.17 | 0.013748 | ENSSSCG00000028758\|ENSSSCG00000010375 |
|  | activin receptor signaling pathway | 0.15 | 0.015729 | ENSSSCG00000015873\|ENSSSCG00000010375 |
|  | negative regulation of tumor necrosis factor production | 0.09 | 0.038319 | ENSSSCG00000000857\|ENSSSCG00000028758 |
|  | G protein-coupled peptide receptor activity | 0.09 | 0.041302 | ENSSSCG00000015320\|ENSSSCG00000001589 |
|  | positive regulation of chemokine production | 0.20 | 0.075359 | ENSSSCG00000028758 |
| **pDC** | positive regulation of kinase activity | 0.28 | 2.29E-06 | ENSSSCG00000003471\|ENSSSCG00000003811\|  ENSSSCG00000006464\|ENSSSCG00000006465 |
|  | calcium ion binding | 0.10 | 1.32E-05 | ENSSSCG00000010426\|ENSSSCG00000014395  \|ENSSSCG00000006981\|ENSSSCG00000002314 |
|  | cell-cell signaling | 0.27 | 0.000663 | ENSSSCG00000021306\|ENSSSCG00000008081  \|ENSSSCG00000004241\|ENSSSCG00000009661 |
|  | positive regulation of MAPK cascade | 0.21 | 0.001293 | ENSSSCG00000006530\|ENSSSCG00000003811  \|ENSSSCG00000006464\|ENSSSCG00000016716 |
|  | cell adhesion | 0.12 | 0.002994 | ENSSSCG00000014395\|ENSSSCG00000001832  \|ENSSSCG00000014324\|ENSSSCG00000028827 |
|  | apoptotic process involved in development | 0.60 | 0.004659 | ENSSSCG00000007238\|ENSSSCG00000015756  \|ENSSSCG00000006191 |
|  | engulfment of apoptotic cell | 0.43 | 0.009313 | ENSSSCG00000007238\|ENSSSCG00000015756  \|ENSSSCG00000006191 |
|  | cell migration | 0.10 | 0.015531 | ENSSSCG00000029612\|ENSSSCG00000029460\|  ENSSSCG00000022726\|ENSSSCG00000026516 |
|  | complement activation, classical pathway | 0.33 | 0.015933 | ENSSSCG00000010077\|ENSSSCG00000005512\|  ENSSSCG00000001422 |
|  | regulation of cell growth | 0.21 | 0.020056 | ENSSSCG00000001382\|ENSSSCG00000022429\|  ENSSSCG00000026978\|ENSSSCG00000000672 |
|  | positive regulation of viral entry into host cell | 0.40 | 0.038567 | ENSSSCG00000024336\|ENSSSCG00000000687 |
| **Mono** | chemokine activity | 0.24 | 1.77E-05 | ENSSSCG00000016254\|ENSSSCG00000008953\|  ENSSSCG00000010966\|ENSSSCG00000017723 |
|  | cellular response to tumor necrosis factor | 0.17 | 3.58E-05 | ENSSSCG00000016254\|ENSSSCG00000008953  \|ENSSSCG00000017723\|ENSSSCG00000017720 |
|  | inflammatory response | 0.09 | 0.00012 | ENSSSCG00000016254\|ENSSSCG00000008090  \|ENSSSCG00000008953\|ENSSSCG00000008087 |
|  | cellular response to interferon-gamma | 0.15 | 0.000784 | ENSSSCG00000016254\|ENSSSCG00000017720  \|ENSSSCG00000017044\|ENSSSCG00000017723 |
|  | calcium ion binding | 0.05 | 0.001356 | ENSSSCG00000027160\|ENSSSCG00000006590  \|ENSSSCG00000022993\|ENSSSCG00000012113 |
|  | monocyte chemotaxis | 0.20 | 0.002227 | ENSSSCG00000017723\|ENSSSCG00000016254\|  ENSSSCG00000017720\|ENSSSCG00000010966 |
|  | cellular response to lipopolysaccharide | 0.10 | 0.004919 | ENSSSCG00000008090\|ENSSSCG00000008953\|  ENSSSCG00000008087\|ENSSSCG00000017044 |
|  | regulation of phagocytosis | 0.40 | 0.010775 | ENSSSCG00000021906\|ENSSSCG00000017178 |
|  | macrophage chemotaxis | 0.33 | 0.014145 | ENSSSCG00000017723\|ENSSSCG00000009477 |
|  | virus receptor activity | 0.25 | 0.022036 | ENSSSCG00000021933\|ENSSSCG00000015886 |
|  | positive regulation of NIK/NF-kappaB signaling | 0.10 | 0.042158 | ENSSSCG00000017178\|ENSSSCG00000010966  \|ENSSSCG00000003251 |

**Supplemental Table S4** Cell-specific genes differentially expressed with polyIC stimulation

| Group | geneID | TAU | log_2_FC | *P* Value | Expression |
| --- | --- | --- | --- | --- | --- |
| Mono | ENSSSCG00000002737 | 0.86333 | 3.91989 | 0.04559 | up |
|  | ENSSSCG00000033918 | 0.93281 | 3.34213 | 0.00284 | up |
|  | ENSSSCG00000004039 | 0.98498 | 3.43664 | 0.02731 | up |
|  | ENSSSCG00000005203 | 0.72737 | 2.75545 | 0.03023 | up |
|  | ENSSSCG00000005222 | 0.96785 | 3.06157 | 0.01593 | up |
|  | ENSSSCG00000005688 | 0.81133 | 3.62059 | 0.03798 | up |
|  | ENSSSCG00000006043 | 0.93245 | 4.16905 | 0.03611 | up |
|  | ENSSSCG00000006988 | 0.89930 | 2.82803 | 0.03371 | up |
|  | ENSSSCG00000007139 | 0.96580 | 3.75150 | 0.02501 | up |
|  | ENSSSCG00000007198 | 0.78465 | -2.30650 | 0.03794 | down |
|  | ENSSSCG00000007448 | 0.89358 | 6.25590 | 0.03522 | up |
|  | ENSSSCG00000008384 | 0.97726 | 4.11432 | 0.02821 | up |
|  | ENSSSCG00000009477 | 0.94919 | 4.08019 | 0.02580 | up |
|  | ENSSSCG00000009869 | 1.00000 | 1.58803 | 0.01599 | up |
|  | ENSSSCG00000011495 | 0.85692 | -2.11890 | 0.03156 | down |
|  | ENSSSCG00000012161 | 0.72548 | -1.97040 | 0.03174 | down |
|  | ENSSSCG00000015301 | 0.81642 | 2.57570 | 0.03037 | up |
|  | ENSSSCG00000016239 | 0.77247 | 4.54422 | 0.04223 | up |
|  | ENSSSCG00000019508 | 0.76374 | 3.46260 | 0.00803 | up |
|  | ENSSSCG00000023054 | 0.92822 | 3.18857 | 0.04904 | up |
|  | ENSSSCG00000033412 | 0.90736 | 2.70964 | 0.01812 | up |
|  | ENSSSCG00000036274 | 0.95166 | 4.32419 | 0.02909 | up |
|  | ENSSSCG00000038929 | 0.72842 | 2.52649 | 0.02881 | up |
|  | ENSSSCG00000045252 | 0.95901 | 2.01505 | 0.02533 | up |
|  | ENSSSCG00000048168 | 0.80216 | 4.37579 | 0.02090 | up |
|  | ENSSSCG00000048309 | 0.90879 | 2.40739 | 0.00882 | up |
|  | ENSSSCG00000051218 | 0.86779 | 2.72999 | 0.01582 | up |
| pDC | ENSSSCG00000051583 | 0.71077 | -1.98798 | 0.02935 | down |
|  | ENSSSCG00000051530 | 0.97468 | -2.00641 | 0.00366 | down |
|  | ENSSSCG00000051419 | 0.92992 | -1.51050 | 0.00001 | down |
|  | ENSSSCG00000051361 | 0.99592 | -2.40091 | 0.00179 | down |
|  | ENSSSCG00000051008 | 0.84134 | -3.29727 | 0.00147 | down |
|  | ENSSSCG00000051005 | 0.87424 | 1.83904 | 0.04277 | up |
|  | ENSSSCG00000050222 | 0.78977 | -1.57807 | 0.04107 | down |
|  | ENSSSCG00000050184 | 0.75035 | -2.71629 | 0.00065 | down |
|  | ENSSSCG00000049674 | 0.79304 | 2.33610 | 0.01721 | up |
|  | ENSSSCG00000049494 | 0.78935 | -2.32198 | 0.00218 | down |
|  | ENSSSCG00000049217 | 0.71394 | -2.77849 | 0.00172 | down |
|  | ENSSSCG00000049113 | 0.99740 | 2.53586 | 0.01347 | up |
|  | ENSSSCG00000049023 | 0.84805 | -3.74135 | 0.00000 | down |
|  | ENSSSCG00000048810 | 0.91692 | -1.56105 | 0.02728 | down |
|  | ENSSSCG00000048255 | 0.78081 | -2.66366 | 0.00010 | down |
|  | ENSSSCG00000047578 | 0.77340 | 2.18103 | 0.01436 | up |
|  | ENSSSCG00000047195 | 0.93917 | 4.10229 | 0.00000 | up |
|  | ENSSSCG00000047084 | 0.91760 | -1.54015 | 0.02471 | down |
|  | ENSSSCG00000046879 | 0.71342 | -1.64874 | 0.03153 | down |
|  | ENSSSCG00000046713 | 0.74148 | 4.37488 | 0.00136 | up |
|  | ENSSSCG00000046396 | 0.85246 | -3.63951 | 0.00032 | down |
|  | ENSSSCG00000046060 | 0.78220 | -1.76412 | 0.02668 | down |
|  | ENSSSCG00000045856 | 0.76768 | 1.56361 | 0.03732 | up |
|  | ENSSSCG00000044931 | 0.70623 | 1.56104 | 0.01272 | up |
|  | ENSSSCG00000044713 | 0.94023 | 1.69600 | 0.02513 | up |
|  | ENSSSCG00000044562 | 0.83654 | -6.28728 | 0.00002 | down |
|  | ENSSSCG00000043786 | 0.98664 | -3.42679 | 0.00011 | down |
|  | ENSSSCG00000043723 | 0.92554 | -1.82880 | 0.03368 | down |
|  | ENSSSCG00000043441 | 0.99235 | -2.72316 | 0.00431 | down |
|  | ENSSSCG00000042268 | 0.75498 | -1.58645 | 0.04953 | down |
|  | ENSSSCG00000042105 | 0.77535 | -2.24014 | 0.00183 | down |
|  | ENSSSCG00000042074 | 1.00000 | -1.55750 | 0.01255 | down |
|  | ENSSSCG00000041801 | 0.96730 | 4.59308 | 0.00005 | up |
|  | ENSSSCG00000041327 | 0.74492 | 2.23124 | 0.01738 | up |
|  | ENSSSCG00000040540 | 0.71476 | 1.53065 | 0.04922 | up |
|  | ENSSSCG00000040492 | 0.96282 | -4.88744 | 0.00000 | down |
|  | ENSSSCG00000040405 | 0.81118 | -2.03011 | 0.00508 | down |
|  | ENSSSCG00000040300 | 0.79385 | -2.75638 | 0.00130 | down |
|  | ENSSSCG00000040019 | 0.86370 | -1.85522 | 0.02913 | down |
|  | ENSSSCG00000039392 | 0.93516 | -2.20379 | 0.00005 | down |
|  | ENSSSCG00000038508 | 0.88373 | -3.53229 | 0.00998 | down |
|  | ENSSSCG00000038071 | 0.78732 | 2.57913 | 0.04228 | up |
|  | ENSSSCG00000038007 | 0.91250 | -1.97550 | 0.02055 | down |
|  | ENSSSCG00000035826 | 0.85317 | 2.34761 | 0.01425 | up |
|  | ENSSSCG00000035489 | 0.75082 | 1.53083 | 0.03796 | up |
|  | ENSSSCG00000035446 | 0.83747 | -1.76520 | 0.00427 | down |
|  | ENSSSCG00000034765 | 0.99821 | -2.96822 | 0.00201 | down |
|  | ENSSSCG00000034708 | 0.90760 | 4.47124 | 0.00011 | up |
|  | ENSSSCG00000033759 | 0.70208 | -2.53729 | 0.01443 | down |
|  | ENSSSCG00000033750 | 0.85151 | 2.04357 | 0.04476 | up |
|  | ENSSSCG00000033721 | 0.87178 | -3.81263 | 0.00000 | down |
|  | ENSSSCG00000033413 | 0.86154 | -1.94147 | 0.02722 | down |
|  | ENSSSCG00000033104 | 0.85342 | -4.03221 | 0.00001 | down |
|  | ENSSSCG00000033043 | 0.77785 | -1.75949 | 0.03007 | down |
|  | ENSSSCG00000032735 | 0.81012 | 1.53820 | 0.02625 | up |
|  | ENSSSCG00000032473 | 0.84688 | -1.73264 | 0.00345 | down |
|  | ENSSSCG00000031955 | 0.75373 | 2.79421 | 0.00607 | up |
|  | ENSSSCG00000031529 | 0.85116 | 3.47656 | 0.00046 | up |
|  | ENSSSCG00000031478 | 0.93371 | 1.78202 | 0.03262 | up |
|  | ENSSSCG00000031149 | 0.84329 | 2.28348 | 0.00664 | up |
|  | ENSSSCG00000030903 | 0.99135 | -3.38132 | 0.01378 | down |
|  | ENSSSCG00000030518 | 0.86381 | -1.58750 | 0.04227 | down |
|  | ENSSSCG00000029796 | 0.88729 | -1.65822 | 0.02723 | down |
|  | ENSSSCG00000029275 | 0.89096 | 2.60923 | 0.02678 | up |
|  | ENSSSCG00000028997 | 0.84683 | -1.54245 | 0.04842 | down |
|  | ENSSSCG00000026145 | 0.85673 | 2.77572 | 0.00077 | up |
|  | ENSSSCG00000025772 | 0.75864 | -2.35714 | 0.01627 | down |
|  | ENSSSCG00000025729 | 0.77394 | -2.35446 | 0.00404 | down |
|  | ENSSSCG00000025687 | 0.77999 | -1.86922 | 0.00109 | down |
|  | ENSSSCG00000025598 | 0.75205 | -2.46797 | 0.00705 | down |
|  | ENSSSCG00000025176 | 0.86433 | -1.59982 | 0.02617 | down |
|  | ENSSSCG00000024288 | 0.86975 | 3.52138 | 0.00965 | up |
|  | ENSSSCG00000023746 | 0.72692 | 3.17100 | 0.00875 | up |
|  | ENSSSCG00000022726 | 0.78901 | -3.10296 | 0.00001 | down |
|  | ENSSSCG00000022140 | 0.99367 | 5.03988 | 0.00005 | up |
|  | ENSSSCG00000021397 | 0.76453 | -1.59740 | 0.02939 | down |
|  | ENSSSCG00000017037 | 0.84684 | 3.67462 | 0.00008 | up |
|  | ENSSSCG00000016763 | 0.98952 | -3.83611 | 0.00001 | down |
|  | ENSSSCG00000016716 | 0.73458 | -2.80500 | 0.01862 | down |
|  | ENSSSCG00000016110 | 0.97120 | 1.72916 | 0.03016 | up |
|  | ENSSSCG00000015802 | 0.76364 | 1.73948 | 0.04867 | up |
|  | ENSSSCG00000015522 | 0.74281 | -2.57040 | 0.00030 | down |
|  | ENSSSCG00000015144 | 0.74431 | -2.05944 | 0.03973 | down |
|  | ENSSSCG00000015035 | 0.94976 | -2.76328 | 0.00472 | down |
|  | ENSSSCG00000015010 | 0.82941 | -1.61218 | 0.01266 | down |
|  | ENSSSCG00000014272 | 0.74675 | -2.98395 | 0.01554 | down |
|  | ENSSSCG00000013760 | 0.74070 | -2.01226 | 0.03191 | down |
|  | ENSSSCG00000013354 | 0.84499 | -1.91173 | 0.04304 | down |
|  | ENSSSCG00000012862 | 0.73555 | -1.59258 | 0.03304 | down |
|  | ENSSSCG00000012564 | 0.76326 | -2.83024 | 0.00405 | down |
|  | ENSSSCG00000011878 | 0.79322 | 6.66548 | 0.00000 | up |
|  | ENSSSCG00000011643 | 0.79377 | 3.62907 | 0.00003 | up |
|  | ENSSSCG00000011622 | 0.83893 | -2.76213 | 0.00581 | down |
|  | ENSSSCG00000011168 | 0.86041 | -2.30431 | 0.00756 | down |
|  | ENSSSCG00000010649 | 0.83000 | 2.91215 | 0.00079 | up |
|  | ENSSSCG00000010272 | 0.85361 | -1.52637 | 0.02437 | down |
|  | ENSSSCG00000009964 | 0.74739 | -1.80908 | 0.03419 | down |
|  | ENSSSCG00000009873 | 0.71159 | -1.84202 | 0.04243 | down |
|  | ENSSSCG00000009748 | 0.93741 | -1.72294 | 0.04265 | down |
|  | ENSSSCG00000009488 | 0.77664 | -3.43142 | 0.00188 | down |
|  | ENSSSCG00000009446 | 0.94316 | 3.88186 | 0.00000 | up |
|  | ENSSSCG00000009194 | 0.84895 | 1.54756 | 0.03564 | up |
|  | ENSSSCG00000009125 | 0.81277 | 3.27982 | 0.01352 | up |
|  | ENSSSCG00000009031 | 0.81874 | 3.22631 | 0.00178 | up |
|  | ENSSSCG00000008923 | 0.70535 | 1.58342 | 0.03027 | up |
|  | ENSSSCG00000008230 | 0.89317 | -2.50463 | 0.00837 | down |
|  | ENSSSCG00000008229 | 0.99370 | -2.14441 | 0.02234 | down |
|  | ENSSSCG00000008122 | 0.84560 | -1.89636 | 0.00122 | down |
|  | ENSSSCG00000007864 | 0.98534 | -1.88502 | 0.00258 | down |
|  | ENSSSCG00000007163 | 0.92974 | -1.59916 | 0.00574 | down |
|  | ENSSSCG00000007116 | 0.92208 | -1.74043 | 2.47563 | down |
|  | ENSSSCG00000007112 | 1.00000 | 6.56674 | 0.00000 | up |
|  | ENSSSCG00000007072 | 0.99465 | 2.72518 | 0.00675 | up |
|  | ENSSSCG00000006393 | 0.80885 | -3.08791 | 0.00207 | down |
|  | ENSSSCG00000006140 | 0.70363 | -3.54544 | 0.00021 | down |
|  | ENSSSCG00000005352 | 0.99547 | -2.09364 | 0.00305 | down |
|  | ENSSSCG00000004776 | 0.89842 | -4.21551 | 0.00000 | down |
|  | ENSSSCG00000004713 | 0.80146 | -2.49052 | 0.00893 | down |
|  | ENSSSCG00000004705 | 0.84494 | -1.83400 | 0.04098 | down |
|  | ENSSSCG00000004540 | 0.85448 | 3.57496 | 0.00420 | up |
|  | ENSSSCG00000004092 | 0.75329 | -3.35636 | 0.00955 | down |
|  | ENSSSCG00000003740 | 0.74081 | -2.27430 | 0.01268 | down |
|  | ENSSSCG00000003646 | 0.70858 | 1.73442 | 0.03307 | up |
|  | ENSSSCG00000003123 | 1.00000 | 3.96550 | 0.00074 | up |
|  | ENSSSCG00000003062 | 0.88301 | 1.52719 | 0.01839 | up |
|  | ENSSSCG00000001650 | 0.87365 | -1.70314 | 0.03376 | down |
|  | ENSSSCG00000001388 | 0.96696 | -2.25747 | 0.00535 | down |
|  | ENSSSCG00000001382 | 0.73574 | -1.91669 | 0.00731 | down |
|  | ENSSSCG00000000940 | 0.77722 | -1.58123 | 0.01731 | down |
|  | ENSSSCG00000000607 | 0.70831 | 2.32245 | 0.03155 | up |
|  | ENSSSCG00000000528 | 0.73473 | -1.91332 | 0.01611 | down |
|  | ENSSSCG00000000517 | 0.78407 | -2.10378 | 0.03701 | down |
|  | ENSSSCG00000000515 | 0.99871 | 2.30727 | 0.02098 | up |
| cDC | ENSSSCG00000001589 | 0.95720 | -3.06620 | 0.00101 | down |
|  | ENSSSCG00000005967 | 0.71210 | 2.28797 | 0.01168 | up |
|  | ENSSSCG00000006051 | 0.73181 | 3.04556 | 0.01500 | up |
|  | ENSSSCG00000006348 | 0.84123 | 2.30741 | 0.04973 | up |
|  | ENSSSCG00000006453 | 0.93244 | 3.43903 | 0.04316 | up |
|  | ENSSSCG00000006572 | 0.74017 | -3.05930 | 0.00004 | down |
|  | ENSSSCG00000009313 | 0.96121 | 3.16733 | 0.02670 | up |
|  | ENSSSCG00000011246 | 0.83416 | -2.49480 | 0.00081 | down |
|  | ENSSSCG00000015632 | 0.95548 | -1.55940 | 0.04607 | down |
|  | ENSSSCG00000016182 | 0.87270 | -2.08910 | 0.00011 | down |
|  | ENSSSCG00000016915 | 0.99103 | 3.86147 | 0.01006 | up |
|  | ENSSSCG00000017062 | 0.72434 | 3.24049 | 0.01526 | up |
|  | ENSSSCG00000017411 | 0.71874 | -2.38310 | 0.00038 | down |
|  | ENSSSCG00000021132 | 0.95110 | 1.56357 | 0.02563 | up |
|  | ENSSSCG00000021515 | 0.79426 | 3.90749 | 0.01102 | up |
|  | ENSSSCG00000022081 | 0.73314 | -3.63590 | 0.00147 | down |
|  | ENSSSCG00000022105 | 0.72767 | -3.10510 | 0.00560 | down |
|  | ENSSSCG00000022473 | 0.78766 | 3.43835 | 0.00099 | up |
|  | ENSSSCG00000022739 | 0.75219 | -2.33710 | 0.01404 | down |
|  | ENSSSCG00000025436 | 0.75437 | 2.08976 | 0.02518 | up |
|  | ENSSSCG00000029334 | 0.97530 | 1.94449 | 0.00168 | up |
|  | ENSSSCG00000036851 | 0.77886 | -1.52880 | 0.04831 | down |
|  | ENSSSCG00000038077 | 0.77476 | -1.73130 | 0.02952 | down |
|  | ENSSSCG00000041446 | 0.90301 | 4.00067 | 0.00000 | up |
|  | ENSSSCG00000046111 | 0.92701 | 3.71547 | 0.00147 | up |
|  | ENSSSCG00000047253 | 0.89049 | 3.51693 | 0.00084 | up |
|  | ENSSSCG00000051406 | 0.72250 | -1.56000 | 0.01052 | down |
